# Knowledge, attitudes and practices of Australian dairy goat farmers towards the control of gastrointestinal parasites

**DOI:** 10.1186/s13071-024-06650-6

**Published:** 2025-01-24

**Authors:** Endris A. Ali, Ghazanfar Abbas, Ian Beveridge, Sandra Baxendell, Berwyn Squire, Mark A. Stevenson, Abdul Ghafar, Abdul Jabbar

**Affiliations:** 1 Melbourne Veterinary School, The University of Melbourne, Werribee, VIC 3030 Australia; 2 Goat Veterinary Consultancies - goatvetoz, Keperra, QLD 4054 Australia; 3 Department of Energy, Environment and Climate Action, Swan Hill, VIC 3585 Australia

**Keywords:** Anthelmintics, Anthelmintic resistance, Dairy goats, Gastrointestinal parasites, *Haemonchus contortus*, Questionnaire

## Abstract

**Background:**

Gastrointestinal parasites such as nematodes and coccidia are responsible for significant economic losses in the goat industry globally. An indiscriminate use of antiparasitic drugs, primarily registered for use in sheep and cattle, in goats has resulted in drug-resistant gastrointestinal parasites. Very little is known about the gastrointestinal parasite control practices used by Australian dairy goat farmers that are pivotal for achieving sustainable control of economically important parasites. The study reported here provides insights into gastrointestinal parasite control practices of Australian dairy goat farmers based on responses to an online survey.

**Methods:**

The questionnaire comprised 58 questions on farm demography, husbandry and grazing management, knowledge of gastrointestinal parasites and their importance in dairy goats, diagnosis of infections, antiparasitic drugs and alternate control options. After a pilot survey (*n* = 15 respondents), a link to the questionnaire was available to all (*n* = 456) registered members of the Dairy Goat Society of Australia Ltd from 17 April to 16 June 2023. Multiple correspondence analyses (MCA) were performed to explore the association between selected parasite control practices.

**Results:**

A total of 66 (14%) respondents completed the questionnaire. Of these, 74% (49/66) observed parasite-related illnesses in their goats; two-thirds of them assessed worms burden using faecal egg counts (FECs), with 26% (39/149) deworming their goats based on the results of the FECs. Most respondents (97%; 183/188) perceived that gastrointestinal parasites caused production losses and ranked *Haemonchus contortus* as the most important parasite. Anitparasitic drugs were used by 94% (62/66) of respondents, with the most frequently used anthelmintics being a commercial combination of four anthelmintics (levamisole, closantel, albendazole and abamectin), benzimidazoles and macrocyclic lactones. Most respondents (77%; 51/66) were unaware of anthelmintic resistance on their property. MCA results delineated two clusters of gastrointestinal parasites management.

**Conclusions:**

This study provides insights into the demography of Australian dairy goat farms, the husbandry and grazing practices used by dairy goat farmers, their knowledge regarding gastrointestinal parasites and their practices for internal parasite control, thereby paving the way for tackling drug resistance in gastrointestinal parasites in dairy goats.

**Graphical Abstract:**

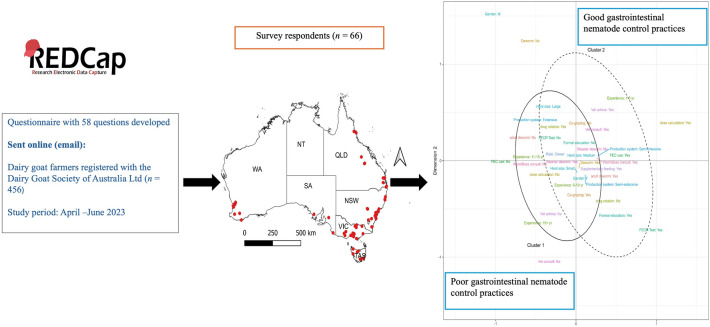

**Supplementary Information:**

The online version contains supplementary material available at 10.1186/s13071-024-06650-6.

## Background

Parasitism is one of the significant constraints to small ruminant production globally as it can cause a reduction in wool growth and quality, milk yield, skeletal growth and live-weight gain [1–5]. Of the various parasites infecting goats, gastrointestinal nematodes (GINs) remain a significant challenge for the goat industry and have been the subject of several reviews [6, 7] as goats can have higher GIN infections than sheep but exhibit lower levels of acquisition and expression of immune responses. Although more than 20 GIN species have been reported in goats worldwide in the last 10 years [8–15], the causes of parasitic gastroenteritis and associated production losses are predominantly associated with *Haemonchus contortus*, *Teladorsagia circumcincta* and *Trichostrongylus* spp. [16, 17]. Other internal parasites of goats, such as *Fasciola hepatica* and *Eimeria* spp., can also lead to economic losses due to relatively high levels of morbidity and mortality. For example, the annual cost of internal parasites, primarily due to GINs, to the Australian goat industry was estimated at 2.54 million Australian dollars (AUD) in 2015 [18] (equivalent to AUD 3.33 million in 2024), with most of these costs attributed to the loss of production. A similar impact is expected to occur in goat industries elsewhere. In the dairy goat industry, these impacts could be even more significant, as GINs lead to a substantial reduction in milk yield [19, 20] and also loss of milk sales due to milk withholding periods after treatment.

Currently, the control of gastrointestinal parasites in goats mainly relies on the indiscriminate use of antiparasitic drugs primarily registered for use in sheep and cattle, resulting in drug resistance in economically important GINs of goats [21]. Recently, Baudinette et al. [22] comprehensively reviewed the literature on anthelmintic resistance (AR) in GINs of goats and found all major nematode genera (i.e., *Haemonchu*s, *Trichostrongylus*, *Teladorsagia*, *Oesophagostomum* and *Cooperia*) to be resistant to commonly used anthelmintics, indicating that AR is becoming a significant issue for the goat industry globally. This situation is further complicated when the off-label use of antiparasitic drugs is common in goats. Current drug pharmacokinetic studies suggest that goats metabolise anthelmintics faster than sheep [23–25]. Moreover, the requirement for registered manufacturers of anthelmintics to provide a milk withdrawal period limits the options available to dairy goat farmers for controlling GINs worldwide.

Owing to the challenges associated with the chemical control of goat parasites, questionnaire surveys have been conducted in several countries to assess parasite control practices used by goat farmers [26–35]. However, knowledge of gastrointestinal parasites, their significance and control practices in dairy goats is limited. For instance, three studies have surveyed Australian goat farmers, mainly meat goat farmers located in two states (i.e. New South Wales and Queensland), to assess parasite control practices [36–38]. In the most recent survey, Brunt et al. [38] reported that 90% (79/88) of goat farmers who completed the survey perceived GINs as an important constraint for the industry and that 85% of these respondents were using anthelmintics to control GINs in goats, with 69% of them having used anthelmintics not registered for use in goats. The majority (93%) of respondents acknowledged AR to be a significant challenge and 25% of them reported the presence of AR on their properties. These findings reflect farmers’ knowledge, practices and attitudes towards parasite control in Australian goats (mainly meat) where production systems are usually extensive and/or semi-extensive. To date, none of the studies have focused on assessing the gastrointestinal parasite control practices on dairy goat farms.

It is worth noting that the production systems, management and husbandry practices of dairy goats significantly differ from those of meat goats, particularly the rangeland goats, and that only one drug, with a milk withholding period, is registered for use in dairy goats against internal parasites in Australia. In the study reported here, we used an online questionnaire to assess gastrointestinal parasite control perceptions and practices of Australian dairy goat farmers. The findings of the study will help identify challenges to achieving sustainable control of goat internal parasites with limited available options.

## Methods

### Dairy goat farms in Australia

The Australian dairy goat industry is one of the emerging livestock industries, producing 16.8 million litres of milk per year with a farm-gate value of AUD 20–27 million [39]. Of the estimated 46,152 dairy goats in Australia, the majority are located in the south-eastern states of New South Wales (NSW), Queensland (QLD) and Victoria (VIC), with smaller numbers in South Australia (SA), Western Australia (WA) and Tasmania (TAS) [39].

The Australian dairy goat industry is still in its infancy compared to the sheep and cattle industries as well as the dairy goat industries elsewhere, and little is known about the national herd's production levels or economic impact. Most goat farmers practice seasonal breeding, as the percentage of pregnancies using artificial breeding is lower (mainly due to technical problems) than natural mating. Although the semi-extensive production system, which combines grazing on pastures supplemented with crop residues and supplementary feed, is the predominant type and increasing, some Australian dairy goat farms run intensive (i.e. usually indoor feeding and commercial dairy goat farming) [39] and semi-intensive (i.e. an intermediate approach involving controlled grazing, with limited access to pasture than semi-extensive system and more regular supplementary feeding) production systems. In either case, improper shed design is common, resulting in high labour costs, suboptimal hygienic conditions and animal health concerns. The primary health constraints are GINs (mainly for pasture-fed goats) [12, 18], Johne’s disease [18], metabolic problems (intensive management with shedding) [40, 41], coccidiosis in young goats [42], kid mortality [43, 44], caprine arthritis encephalitis [18, 45, 46], mastitis [47] and foot problems [48].

### Questionnaire

A questionnaire survey was conducted using an online software tool, Research Electronic Data Capture (REDCap) (www.project-redcap.org). The questionnaire (Additional file 1: Text S1) consisted of 58 questions stratified into five themes, including: (i) farm and respondent demography (number of questions, *n* = 9); (ii) husbandry and grazing management (*n* = 15); (iii) knowledge about gastrointestinal parasites and their importance (*n* = 8); (iv) diagnosis of gastrointestinal parasites (*n* = 6); and (v) antiparasitic drugs and control methods (*n* = 15). Respondents were also asked specific questions (*n* = 5) about coccidiosis. Most questions (*n* = 52) were close-ended, with multiple choices, Likert scale or yes/no options. For most close-ended questions (*n* = 25), an additional category of ‘other’ was included if the respondents had a different answer. Six open-ended questions allowed respondents to provide descriptive information or numerical values. Branching logic was used for questions that were interconnected or linked to each other, guiding respondents based on their previous answers. The age of goats was categorised into three groups: kids (up to 6 months), weaners (> 6 months to 1 year) and adults (> 1 year). Goats were further categorised into kids (up to 6 months), weaners (> 6 months to 1 year), milkers/does and bucks for questions on the number of goats on farms and the frequency of changing bedding. Based on the total number of goats in each age category, herd size was categorised as small (< 20 goats), medium (20–100 goats) and large (> 100 goats).

Dairy goat farms registered with the Dairy Goat Society of Australia Ltd (DGSA) constituted the source population for the survey and their participation in the study was entirely voluntary. The pilot survey was conducted with 15 dairy goat farmers and these data were excluded from the final analyses. The feedback received from the pilot survey was incorporated into the final survey. Subsequently, registered members (*n* = 456) of DGSA Ltd were invited (via email) to participate in the survey on 17 April 2023. The email included a link to the online questionnaire provided by the DGSA’s Ltd head office. Furthermore, the survey link was also sent to their members by state branches of the DGSA Ltd. Social media platforms such as Twitter and Facebook were also used to promote the survey. The DGSA's Ltd head office sent five reminder emails to all members. The questionnaire remained accessible to Australian dairy goat farmers until 16 June 2023 (2 months). Participants were able to complete the survey anonymously. This anonymity was important as legislation in many Australian states does not allow worm treatments at higher-than-label dose rates or for sheep or cattle worm treatments for goats without a veterinary prescription.

### Data analyses

Questionnaire data were downloaded from REDCap as a comma separated value file with 115 responses recorded, followed by manual checking of the completeness and implausible values of the data using Microsoft Excel® version 2016 (Microsoft Corp., Redmond, WA, USA). The final dataset comprised questionnaire responses that were either fully completed (*n* = 68) as shown by REDCap or partially completed, with > 50% of questions answered (*n* = 5). After thorough error checks and a review of free text comments, data were coded into additional categories where necessary. Open-ended questions with ‘other’ options were merged or reclassified as appropriate. The patterns of ‘missingness’ in the data were imputed by the multiple imputation method using the contributed MICE package in R software [49]. Missing values for all the categorical and continuous questionnaire response variables were imputed using a classification and regression tree method. Following imputation, descriptive analyses were conducted for each question, generating frequencies for categorical variables and calculating means and medians for continuous variables. The percentage of responses was determined by dividing the number of responses by the total number of survey respondents. In cases where questions allowed for multiple answers, the denominator's total exceeded the overall survey respondent count.

The proportional similarity index (PSI) was used to assess the representativeness of the participating farms in the dairy goat industry of Australia [50]. The PSI quantified the agreement in the frequency distribution of dairy goat farms that responded to the survey, stratified by state, with the frequency distribution of total dairy goat farms registered with DGSA Ltd in six states of Australia.

Multiple correspondence analysis (MCA) was conducted to identify underlying structures among survey respondents [51] based on the selected GIN control practices in goats recommended by WormBoss https://wormboss.com.au (Additional file 2: Table S1). Cluster analysis was performed using hierarchical clustering on principal components (HCPC) using Ward’s criterion to identify clusters of respondents who reported either ‘good’ (optimal) or ‘poor’ (suboptimal) GIN control practices. The contributed FactoMine R package [52] was utilised to perform MCA and HCPC in R.

**Fig. 1:**
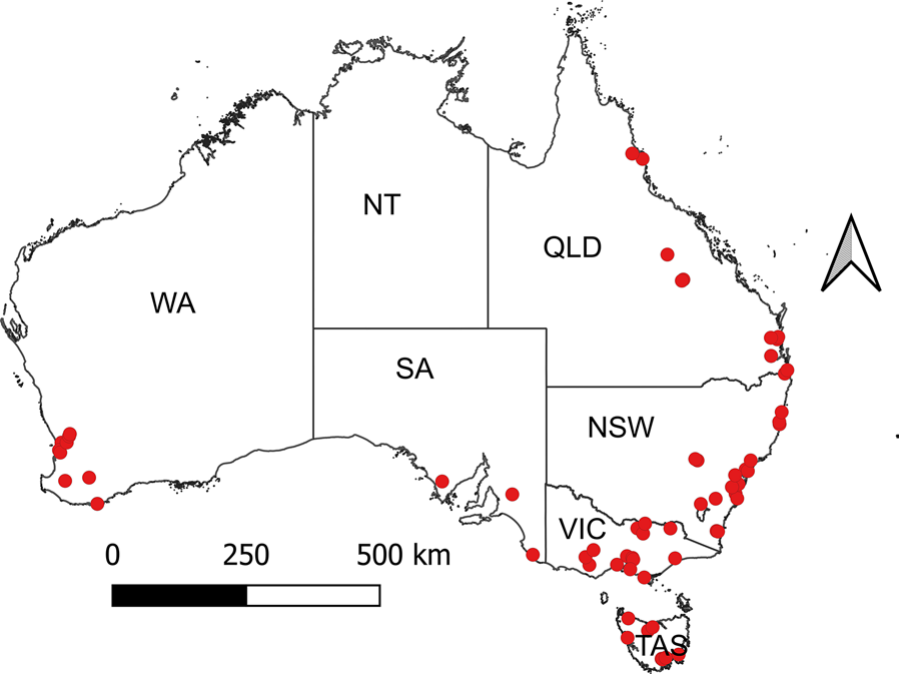
Map of Australia showing the distribution of dairy goat farmers that responded to the survey. Each participating farm is shown as a red dot. States and territories of Australia: NSW, New South Wales; NT, the Northern Territory; QLD, Queensland; SA, South Australia; TAS, Tasmania; VIC, Victoria; WA, Western Australia

All analyses and data visualisation procedures were performed using R version 4.3.1 [53] and GraphPad Prism version 10.3.0 for Windows (www.graphpad.com). The QGIS 3.34 software package was used to generate a map showing the postcode area of survey respondents (see Fig. 1).

## Results

### General characteristics of dairy goat farms

A total of 115 farmers accessed the survey invitation link, resulting in 68 completed and 47 incomplete responses. After appraising the incomplete responses, five fulfilled the pre-set inclusion criteria (i.e. addressed main questions on farm demographics, use of diagnostics and administration of antiparasitic drugs) and were included in our data analyses. Seven respondents were meat/cashmere goat farmers and were, therefore, excluded from further analysis, leaving 66 eligible responses for the final analysis. The response rate for the questionnaire was 14% (66 of 456).

**Fig. 2:**
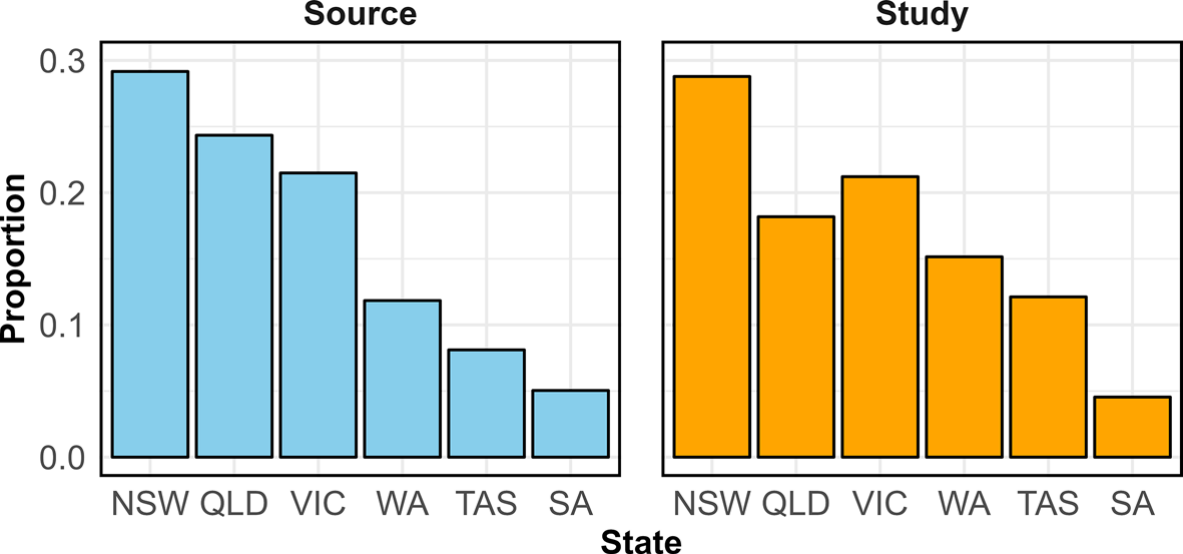
Bar plots showing the relative frequencies of the source and study populations stratified by states of Australia. NSW, New South Wales; QLD, Queensland; SA, South Australia; TAS, Tasmania; VIC, Victoria; WA, Western Australia

Of the 66 respondents included in the final analysis, the highest number was from NSW (29%) followed by VIC (21%), QLD (18%), WA (15%), TAS (12%) and SA (5%). The similarity of frequencies between the total dairy goat farms registered with DGSA Ltd and those that responded to the survey was 93% (range 75–94%). The relative frequencies of the respondents (study population) were consistent with those of dairy goat farmers registered with DGSA Ltd (Fig. 2). Most respondents had a medium-sized herd of goats (56%) followed by small (36%) and large (8%) herds. Overall, the mean number of goats on respondents farms was 69 (range 3–1428), with kids (mean 27; range 0–700), milkers/does (27; range 0–600) and weaners (11; range 0–200) the most common age groups (Table 1).

**Table 1:** Demographic information on Australian dairy goat farms (*n* = 66) reported by Australian goat farmers who responded to survey

Question	Mean	Median (Q1, Q3)	Min, Max
Farm area (acres)	76	17 (8, 39)	0.75, 2,000
Grazing area (acres)	56	10 (4, 20)	0.25, 2,000
Number of goats in the last year	69	26 (16, 42)	3, 1,428
- Kids (≤ 6 months)	27	8 (5, 15)	0, 700
- Weaners (> 6 months to 1 year)	11	4 (3, 10)	0, 200
- Milkers/does	27	9 (5, 15)	0, 600
- Bucks	4	3 (1, 4)	0, 30

*Max* Maximum, *Min* minimum, *Q1* first quartile, *Q3* third quartile

The mean total area of the 66 farms was 76 (range 0.75–2000) acres, with a mean of 56 (range 0.25–2000) acres allocated to grazing area (Table 1). Most of the respondents were female (91%; 60/66), owned their farms (94%), had no formal qualifications (80%) and had 1 to 10 years of experience working in the dairy goat industry (62%) (Table 2). Among the qualified respondents (20%; 13/66), 39% (5/13) and 31% had a certificate or a diploma in farm animal management, respectively. Most farms had been engaged in dairy goat farming for 1 to 5 years (38%; 25/66) (Table 2).

**Table 2:** Demographic information on Australian dairy goat farmers (*n* = 66) who responded to the survey

Question	Levels	Percentage (counts)
Role on the farm	Farm owner	94 (62)
	Farm manager	5 (3)
	Staff worker	1 (1)
Gender	Female	91 (60)
	Male	8 (5)
	Prefer not to disclose	1 (1)
Experience in goat farming (years)	1–5	38 (25)
	6–10	18 (12)
	11–15	12 (8)
	> 15	32 (21)
Use of property for goat farming (years)	< 1	3 (2)
	1–10	62 (41)
	11–20	18 (12)
	21–30	5 (3)
	31- 40 > 40	8 (5) 5 (3)
Formal qualification	No	80 (53)
	Yes	20 (13)
Type of qualification (*n* = 13)	Certificate in farm animals	39 (5)
	Diploma in farm animals	31 (4)
	Veterinary nursing	15 (2)
	Bachelor’s degree or above	15 (2)

### Husbandry and grazing management

**Fig. 3:**
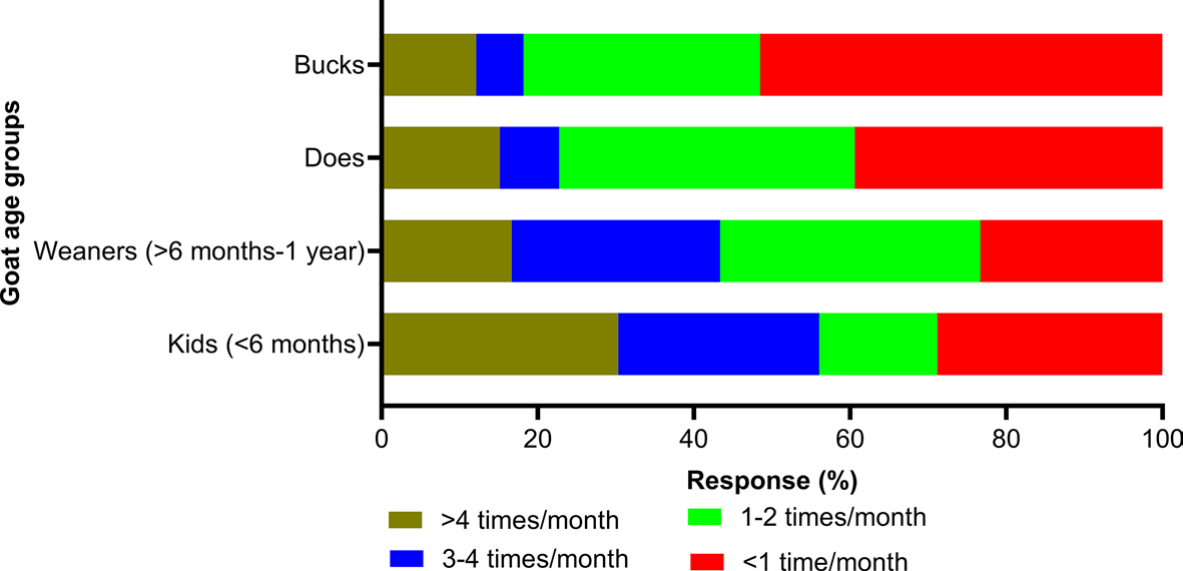
Percentage of respondents (*n* = 66) reporting the frequency of changing bedding material in goat pens at Australian dairy goat farms

The main goat breeds kept by the dairy goat farmers were Anglo-Nubian (25%; 35/140), Nigerian Dwarf (16%), Saanen (14%) and Toggenburg (14%) (Table 3). Most of the respondents (65%, 43/66) were using a semi-extensive goat production system, with the average covered area allowance per housed goat > 3.0 m^2^ (39%; 26/66). Although spring was the main kidding season on most farms (64%; 42/66), it also occurred in other seasons, and weaning age was > 10 weeks on the majority of farms (86%; 57/66) (Table 3). Most respondents (76%, 50/66) cleaned pens before each kidding (Additional file 3: Figure S1). Wheat straw or wood shavings on solid floors were the most commonly used bedding material (55%; 38/69) for indoor housing of does (Table 3), with the majority of respondents changing bedding material less than 1 time/month and/or 1–2 times/month for all age groups of goats (Fig. 3). Dairy goats were raised with other livestock by 85% (93/109) of respondents, including cattle (25%; 27/109), sheep (20%) and equines (15%). For shared paddocks (58%; 38/66), rotational or co-grazing practices were common (*n* = 48), particularly with cattle (29%; 20/70), horses (26%) and sheep (20%) (Table 3).

**Table 3:** Husbandry and grazing management practices on Australian dairy goat farms reported by Australian dairy goat farmers who responded to the survey

Question (responses)	Levels	Percentage (counts)
Breed (*n* = 140)^a^	Anglo-Nubian	25 (35)
	Nigerian Dwarf	16 (22)
	Saanen	14 (20)
	Toggenburg	14 (19)
	British Alpine	9 (12)
	Alpine	4 (6)
	Australian Melaan	3 (4)
	Lamancha	2 (3)
	Australian Brown	1 (2)
	Sable	1 (2)
	Swiss dairy	1 (1)
	Other^b^	10 (14)
Production system (*n* = 66)	Semi-extensive	65 (43)
	Extensive	23 (15)
	Semi-intensive	12 (8)
Average covered area space allowance/housed goat (*n* = 66)	More than 3.0 m^2^/goat	39 (26)
	2.0–2.5 m^2^/goat	17 (11)
	1.0–1.5 m^2^/goat	15 (10)
	1.5–2.0 m^2^/goat	12 (8)
	0.5–1.0 m^2^/goat	9 (6)
	2.5–3.0 m^2^/goat	5 (3)
	< 0.5 m^2^/goat	3 (2)
Bedding material for indoor housing of does (*n* = 69)^a^	Solid floor with wheat straw or wood shaving	61 (42)
	Dirt floor	27 (19)
	Slatted floor with plastic, wood or extended metal	9 (6)
	None (indoor housing not available)	3 (2)
Kidding season (*n* = 66)	Spring	64 (42)
	Winter	23 (15)
	Autumn	6 (4)
	Summer	5 (3)
	Non-seasonal	3 (2)
Average weaning age (*n* = 66)	> 10 weeks	86 (57)
	8–10 weeks	14 (9)
Keeping goats with other livestock species (*n* = 109)^a^	Cattle	25 (27)
	Sheep	20 (22)
	Horses	15 (16)
	Alpacas/camels	12 (13)
	Birds	9 (10)
	Pigs	5 (5)
	None	15 (16)
Share paddocks (*n* = 66)	No	42 (28)
	Yes	38 (25)
	Occasionally	20 (13)
Grazing practice of farms when paddocks shared (*n* = 48)^a^	Co-grazing with other livestock species	60 (29)
	Rotational grazing	40 (19)
Other livestock species grazing with goats (*n* = 70)^a^	Cattle	29 (20)
	Horses	26 (18)
	Sheep	20 (14)
	Alpacas/camels	16 (11)
	Other	10 (7)
Age category of cattle grazing with goats (*n* = 20)	Cattle between 1–2 years	35 (7)
	Cattle > 2 years	30 (6)
	Weaned calves < 1 year	20 (4)
	Unweaned calves	15 (3)

^a^These questions had more than one answer

^b^ Cashmere, Boer, Australian miniature or Pygmy

Fresh colostrum collected from does kept on the farm was the main source of colostrum for kids (78%; 63/81), with bottle feeding (47%; 53/113) and natural suckling (41%) being the main methods kids were fed. The main feeds used for weaners were roughage (37%; 66/179) and concentrates (35%) while those for does were roughage and concentrate (each 37%; 64/175) and browsing and grazing (17%) (Additional file 3: Table S1). Most of the respondents (95%; 63/66) provided supplementary feed to goats, particularly for the provision of additional energy for lactating does (44%; 48/110), due to poor quality of pasture (18%) or insufficient feed on offer (17%), and 76% (50/66) of the respondents had no access to surface water other than that provided artificially (Additional file 2: Table S2).

### Knowledge of gastrointestinal parasites

**Fig. 4:**
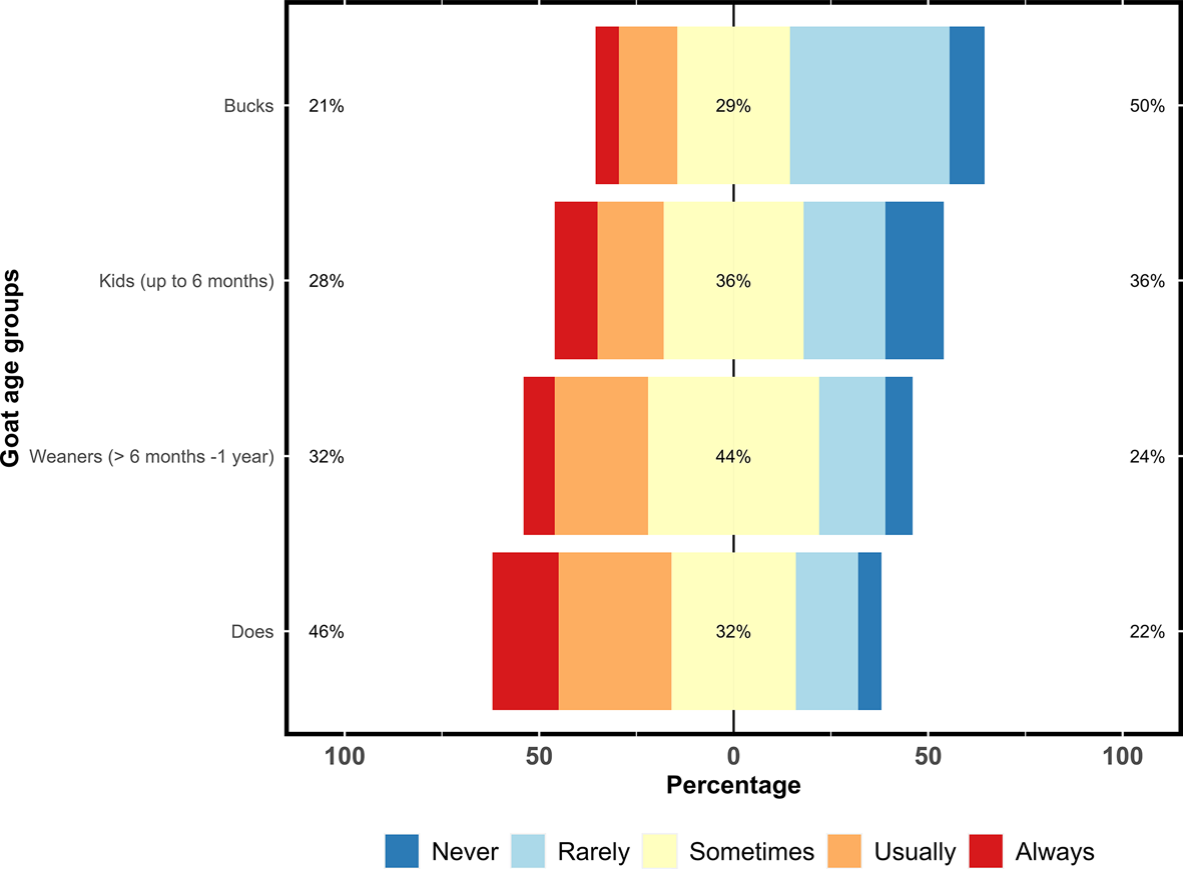
Percentage of respondents (*n* = 66) reporting their perceptions on the risk of gastrointestinal parasites in different age groups of Australian dairy goat farms using a Likert scale. The figure utilises a 5-point Likert scale, allowing respondents to express their perceptions of the risk of gastrointestinal parasites in different age groups of Australian dairy goat farms. The scale ranges from ‘always’ (strong) agreement to ‘never’ (strong disagreement)

The majority of the respondents (74%; 49/66) had observed parasite-related illnesses in their goats based on clinical signs such as scouring/diarrhoea and/or perineal soiling (20%; 47/241), anaemia (17%), rough hair coat (15%), weight loss (14%), high FAMACHA© score (13%), bottle jaw (9%), weakness (6%) and death (4%). Furthermore, the respondents observed parasite-related illnesses/production losses, primarily in spring (32%; 34/105) followed by autumn (19%), summer (18%) and winter (12%). Does/milkers (46%; 30/66) and weaners (32%) were always and/or usually perceived to be susceptible to gastrointestinal parasites (Fig. 4).

**Fig. 5:**
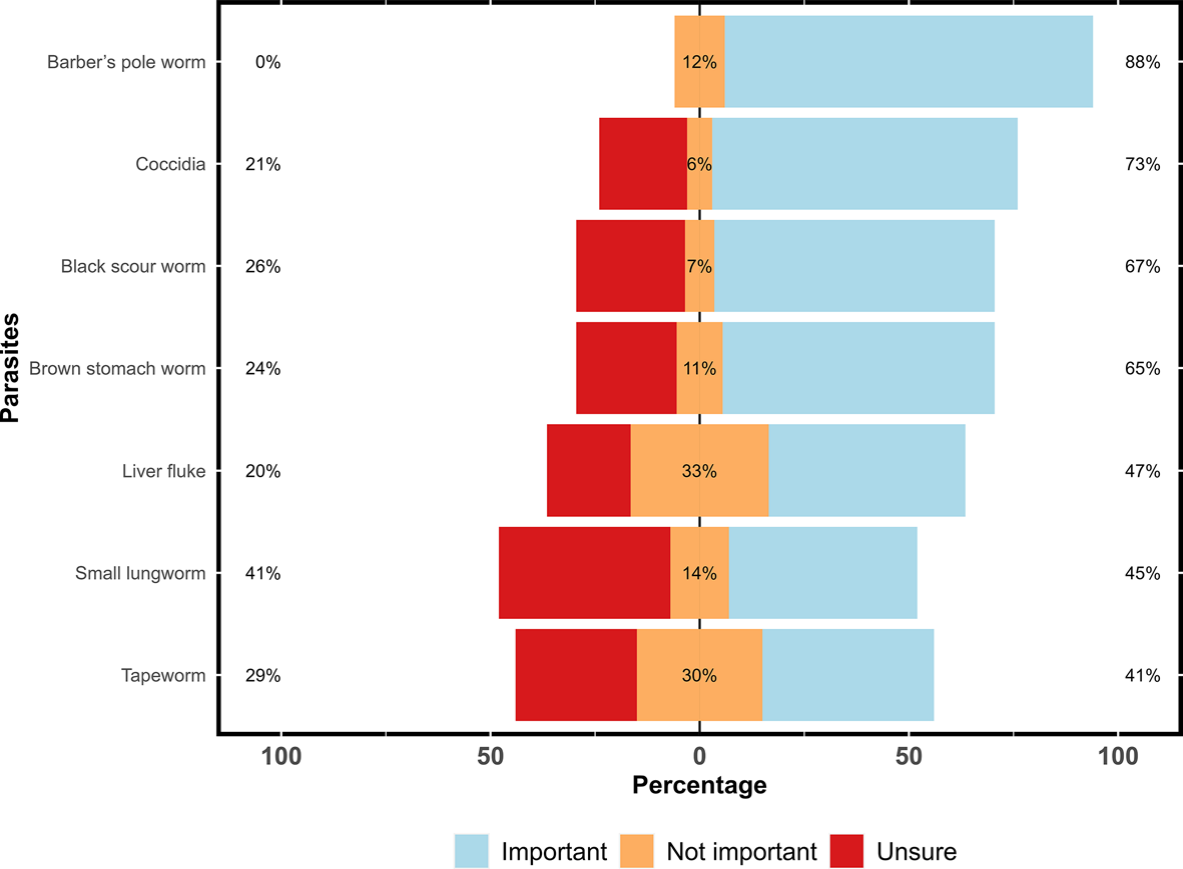
Percentage of respondents (*n* = 66) reporting their perceptions about the occurrence of various gastrointestinal parasites in Australian dairy goat farms using a Likert scale. The figure uses a 3-point Likert scale that allows dairy goat farmers to report their perceptions of the occurrence of various gastrointestinal parasites on their farms. The scale ranges from ‘important’ (strong agreement) to ‘not important’ (strong disagreement), with a neutral option (‘unsure’)

**Fig. 6:**
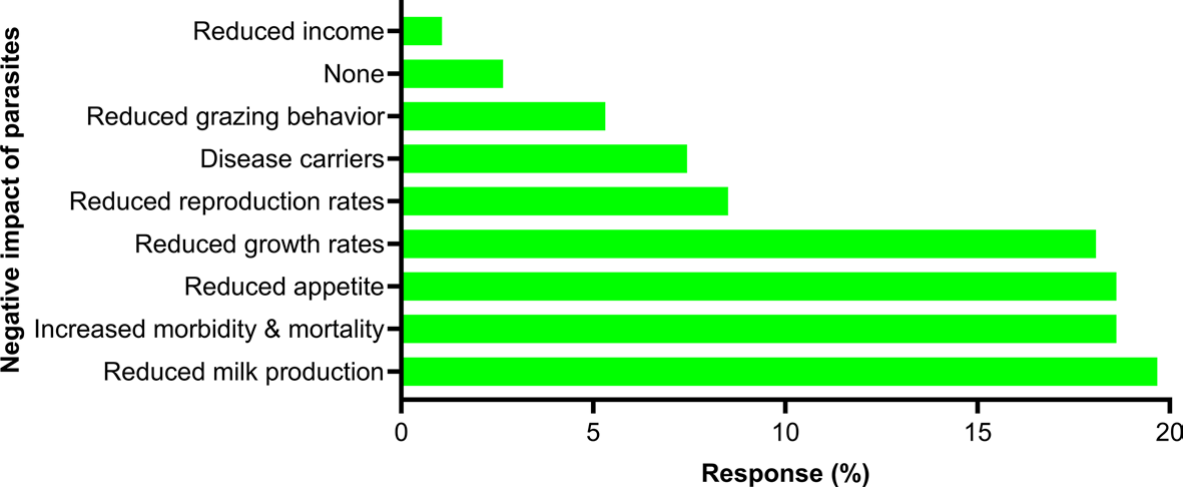
Percentage of respondents (*n* = 66) reporting their perceptions of the impact of gastrointestinal parasites on their dairy goats in Australia

Of the 66 respondents, 88% (*n* = 58) and 73% (*n* = 48) ranked the barber’s pole worm (*H. contortus*) and coccidia (*Eimeria* spp.), respectively, as the two important parasites of dairy goats. Among other gastrointestinal parasites, black scour worm (*Trichostrongylus* spp.) (67%; 44/66), brown stomach worm (*T. circumcincta*) (65%), liver fluke (*F. hepatica*) (47%), small lungworm (*Muellerius capillaris*) (45%) and tapeworm (*Moniezia* spp.) (41%) were also considered important for goat health and production (Fig. 5). Based on the diagnosis of gastrointestinal parasites, 28% (51/180) of the respondents believed that barber’s pole worm was the most commonly diagnosed parasite in their animals followed by black scour worms (16%) and coccidia (15%) (Additional file 3: Figure S2). The respondents believed that the most significant impact of gastrointestinal parasites on their goats was a decrease in milk production (20%; 37/188) followed by higher morbidity and mortality (19%), anorexia (19%) and reduced growth (18%) and reproductive rates (9%) (Fig. 6).

Respondents perceived that contamination of feed with faecal material (28%; 30/106), over-grazing (21%), higher stocking density (19%), suboptimal hygienic conditions (8%), incorrect use of antiparasitic drugs (5%), malnutrition (1%) and other factors (19%) were the most common risk factors for the transmission of gastrointestinal parasites in dairy goats. Those respondents who selected the option of ‘other’ (20/106) thought that wet weather and/or events such as goat shows (9/20), a lack of registered antiparasitic drugs for dairy goats and AR (3/20), grazing pasture (½0), goat kids on the farm (½0), wild/feral animals in paddocks (½0) and green feed in paddocks with warm moist conditions (½0) were other plausible factors contributing to the transmission of gastrointestinal parasites.

### Diagnosis of gastrointestinal parasites

Approximately 55% (36/66) of respondents sought veterinary advice to diagnose and treat suspected gastrointestinal parasite-related illnesses in their goats. Respondents diagnosed goat parasites based on faecal egg counts (FECs) (33%; 51/155), clinical signs (32%), larval culture (14%) or the observation of parasites in faeces (12%) (Table 4). The majority of respondents (67%; 44/66) used FECs for the monitoring of worms burden (27%; 41/149), deworming decisions (26%), assessing the efficacy of anthelmintics (25%) or diagnosing illness suspected of parasitism (21%). FECs were performed either on the farm (42%; 28/66) or at a diagnostic laboratory (40%), with faecal samples being collected from all groups of goats, including does/milkers (21%; 42/196), weaners (20%) and bucks (18%) (Table 4).

**Table 4:** Knowledge of the diagnosis of gastrointestinal parasites known by Australian dairy goat farmers who responded to the survey

Question (responses)	Levels	Percentage (counts)
Veterinary advice sought (*n* = 66)	Yes	55 (36)
	No	46 (30)
Diagnostic method(s) used (*n* = 155)^a^	Faecal egg counts	33 (51)
	Clinical signs	32 (50)
	Larval culture	13 (21)
	Observation of worms in the faeces	12 (19)
	Post-mortem examination	5 (7)
	Haematological examination	2 (3)
	FAMACHA©/anaemia	2 (3)
	None	1 (1)
Use of FEC (*n* = 66)	Yes	67 (44)
	No	32 (21)
	Do not know	2 (1)
Aim of FEC (*n* = 149)^a^	Monitoring of worms burden	28 (41)
	Deworming decisions	26 (39)
	Assessing the efficacy of dewormers	25 (37)
	Diagnosing illness due to worms	21 (31)
	Testing new goats in quarantine	1 (1)
Location of FEC testing (*n* = 66)	On-farm by the staff	42 (28)
	Diagnostic laboratory	40 (26)
	Veterinary clinic	18 (12)
Goats tested for FEC (*n* = 196)^a^	Kids (≤ 6 months)	16 (31)
	Weaners (> 6 months – 1 year)	20 (39)
	Does/milkers	21 (42)
	Wethers	13 (26)
	Bucks	18 (36)
	Aged (> 6 years)	11 (22)

*FAMACHA©* FAffa MAlan CHArt (a selective approach for* H. contortus*), *FEC* faecal egg count

^a^These questions had more than one answer

### Antiparasitic drugs and other control methods for gastrointestinal parasites

**Fig. 7:**
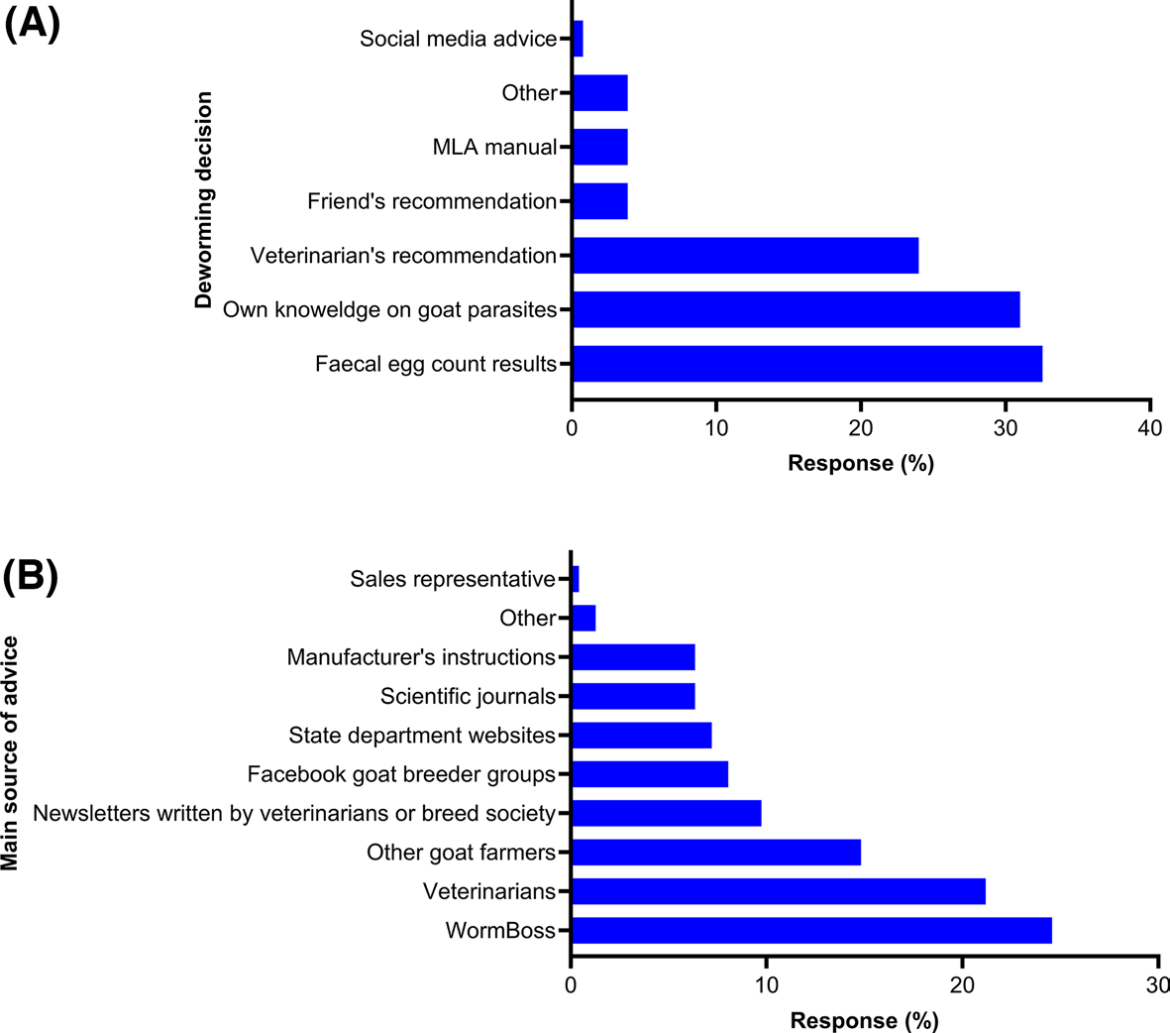
Percentage of respondents (*n* = 66) reporting their perceptions of decision-making (**A**) and sources of advice (**B**) for controlling gastrointestinal parasites in Australian dairy goats. MLA, Meat and Livestock Australia

**Fig. 8:**
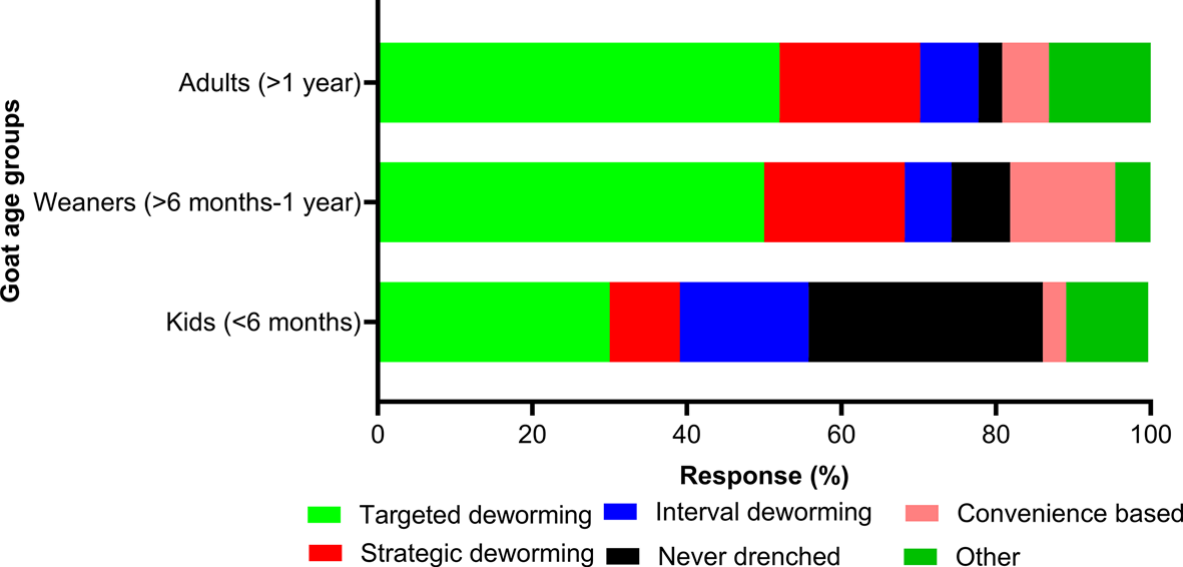
Percentage of respondents (*n* = 66) reporting their perceptions on the selection of deworming strategies at Australian dairy goat farms

The overwhelming majority of respondents (94%; 62/66) used antiparasitic drugs via oral (69%; 49/71) or injectable (10%) routes to control parasitism in their goats (Table 5). The use of antiparasitic drugs was mainly based on FECs (33%; 42/129), respondents’ knowledge of goat parasites (31%) or veterinarians’ recommendations (22%) (Fig. 7a). The main sources of advice on the use of antiparasitic drugs were WormBoss (25%; 58/236 [https://wormboss.com.au]), veterinarians (21%) and other goat farmers (15%) (Fig. 7b). A targeted deworming strategy was used for weaners (52%; 34/66), adults (50%) and kids (30%) (Fig. 8). While 38% (48/128) of the dairy goat farmers did not follow any deworming schedule (i.e. month or season), some indicated a preference for summer (16%; 21/128), autumn (16%), winter (14%) and spring (20%) (Table 5).

**Table 5:** Antiparasitic drugs used to control gastrointestinal parasites reported by Australian dairy goat farmers who responded to the survey

Question (responses)	Levels	Percentage (counts)
Use antiparasitic drugs (*n* = 66)	Yes	94 (62)
	No	6 (4)
Drug class(es) used (n = 177)^a^		
Benzimidazoles (*n* = 42)	Fenbendazole^b^	83 (35)
	Albendazole	14 (6)
	Non-specified BZs	2 (1)
Macrocyclic lactones (*n* = 40)	Ivermectin	40 (16)
	Doramectin	30 (12)
	Abamectin	28 (11)
	Moxidectin	3 (1)
Other (*n* = 19)	Levamisole	26 (5)
	Toltrazuril	26 (5)
	Closantel	21 (4)
	Morantel citrate	21 (4)
	Monepantel	5 (1)
Commercial combination(s) (*n* = 76)	4 (BZs + MLs + LEV + Closantel)	59 (45)
	2 (MLs + Monepantel) 2 (MLs + Derquantel)	24 (18) 7 (5)
	3 (BZs + MLs + LEV)	7 (5)
	2 (BZs + LEV)	2 (2)
	2 (MLs + PZQT)	1 (1)
Preparation(s) of antiparasitic drugs used (*n* = 71)^a^	Oral	69 (49)
	Injectable	10 (7)
	Oral and injectable	8 (6)
	Oral and pour on	6 (4)
	Oral, injectable and pour on	4 (3)
	Pour on	3 (2)
Season(s) of dewormer (*n* = 128)^a^	No fixed schedule	38 (48)
	Spring	20 (26)
	Autumn	16 (21)
	Summer	16 (21)
	Winter	14 (18)
Rotating/changing antiparasitic drug(s) (*n* = 66)	No rotation	45 (30)
	Annually	30 (20)
	Every two years	23 (15)
	Every three years	2 (1)

*BZ* Benzimidazoles, *LEV* levamisole, *MLs* macrocyclic lactones, *PZQT* praziquantel

^a^These questions had more than one answers

^b^The only anthelminthic drug currently registered for dairy goat use in Australia

The choice of selecting various antiparasitic drugs varied among farms but it was dominated by a combination of four anthelmintics (abamectin, albendazole, levamisole and closantel) (25%; 45/177) followed by benzimidazoles (BZs; 24%) and macrocyclic lactones (MLs; 23%) (Additional file 3: Figure S3; Table 5). Among the BZ and ML classes, fenbendazole (83%; 35/42) and ivermectin (40%; 16/40) were the most frequently used single-active products, respectively (Table 5). Approximately 55% (36/66) of the respondents rotated antiparasitic drugs, with 30% of them rotating drugs every year (Table 5). In addition to the use of antiparasitic drugs, BioWorma® (58%; 43/76), copper oxide wire particle (COWP) boluses (22%), and the vaccine Barbervax® (4%) were also used to control GINs of goats, and almost one-fifth of the respondents (16%) used herbal medications such as garlic and a herbal tonic manufactured by AJ Products in South Australia (https://ajproducts.com.au).

**Fig. 9:**
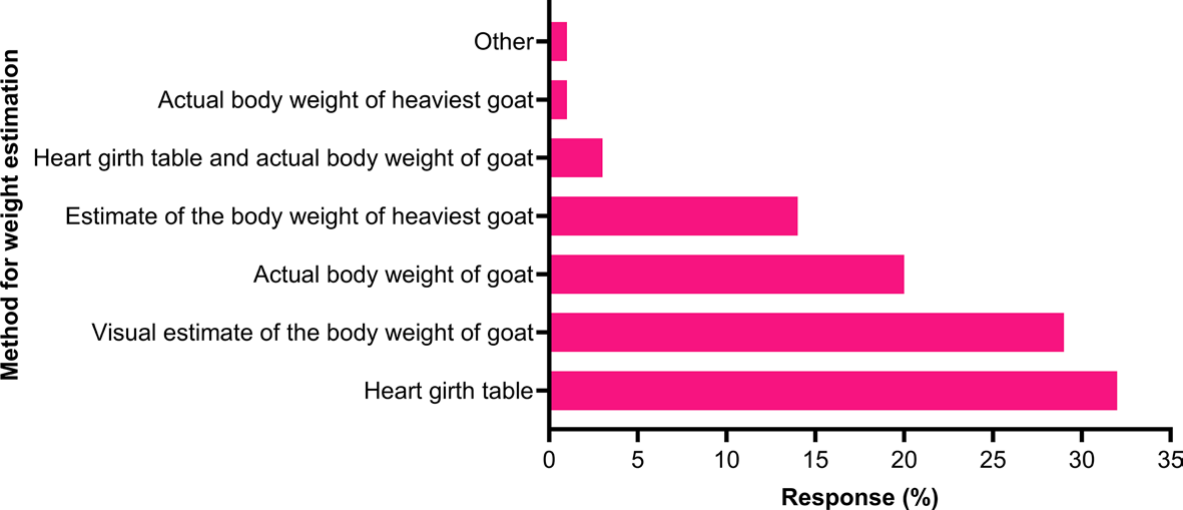
Percentage of respondents (*n* = 66) reporting the criteria used for estimating the weight of dairy goats to calculate the dose of anthelmintics

Half of the respondents (33/66) reported the first use of antiparasitic drugs at weaning or pre-weaning followed by based on diagnosis (FECs or suspected parasites-related illnesses) (32%) and post-weaning (12%). Although most of the dairy goat farmers (53%; 35/66) used 1.5-fold the dose rate recommended for sheep, they did not use a drenching gun (52%; 34/66) and when a drenching gun was used, they did not check its accuracy (5%). Additionally, a significant number of farmers used anthelmintics at two fold the sheep dose (12%; 8/66), and onefold the sheep dose (10%), 2.5- to three fold the sheep dose (8%), with 17% respondents selecting ‘other option’ (e.g. based on the veterinarian prescription). The dose of antiparasitic drugs was calculated based on the weight of goats measured using a heart girth table (32%; 21/66), visual estimation of each goat (29%) and the actual body weight of the goats (20%) (Fig. 9). Most respondents (77%; 51/66) were unaware of AR to different anthelmintics on their properties. Only 23% had tested for AR using FEC reduction tests (FECRTs), and all reported that fenbendazole was ineffective on their farms.

### Multiple correspondence and principal component analyses

**Fig. 10:**
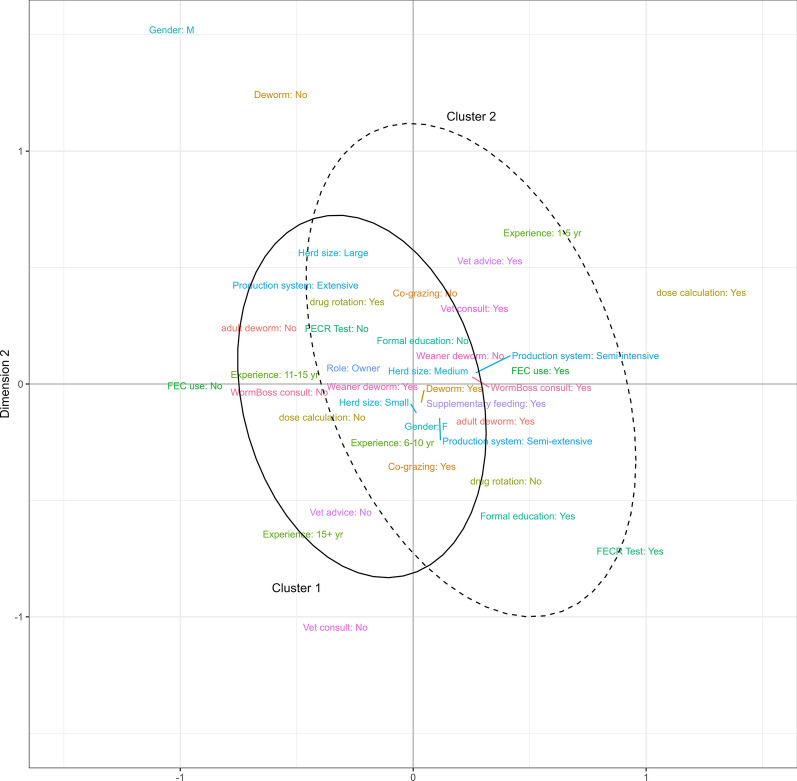
A biplot illustrating the results of a multiple correspondence analysis and hierarchical clustering on principal components analysis, for best GIN control practices in Australian dairy goat farms. Two different clusters are identified: cluster 1, representing ‘poor GIN control practices’, and cluster 2, representing ‘good GIN control practices’. The location of variables along the two dimensions reflects their relationships, with closer proximity indicating stronger associations between practices. FEC, Faecal egg count; GIN, gastrointestinal nematode

The MCA results of the selected GIN control practices across the first two dimensions indicated notable variation, with both dimensions collectively accounting for 20% of the variance (Fig. 10). The position of variables along these dimensions reflects their relationships, with closer proximity on either axis of the biplot indicating stronger associations between practices. The superimposed ellipses derived from HCPC analysis classified respondents into two groups, namely cluster 1 and cluster 2, representing ‘poor’ and ‘good’ GIN control practices, respectively. Respondents in cluster 1 were mainly farm owners/managers operating extensive production systems with 11–15 years or more of dairy goat farming experience. These respondents were less likely to seek veterinary advice for diagnosing and treating suspected gastrointestinal parasite-related illnesses and did not routinely calculate proper dewormer dosages for their goats. In contrast, respondents in cluster 2 managed semi-extensive or semi-intensive production systems and had 1–5 years of dairy goat farming experience. They were seeking veterinary advice for diagnosing and treating suspected gastrointestinal parasite-related illnesses in their goats along with performing FECs and FECRTs.

### Coccidiosis

Almost half of the respondents (52%; 34/66) reported the presence of coccidiosis on their farms. The main clinical signs were observed in kids up to 8 weeks, including diarrhoea and/or perineal soiling, reduced weight gain and tenesmus (Additional file 3: Figure S4). The most commonly used anticoccidial drug was toltrazuril (85%; 29/34) while only 47% (16/34) used coccidiostats for kids (81%; 13/16) or weaners (19%).

## Discussion

This is the first comprehensive study to assess the knowledge and perceptions of dairy goat farmers on parasite control practices in Australia. Approximately 74% (49/66) of farmers who responded had observed gastrointestinal parasite-related illnesses in their goats and two-thirds (49/66) assessed worm burden using FECs. Almost all respondents (97%) perceived that gastrointestinal parasitic infections caused production losses, resulting in widespread use of antiparasitic drugs (94%; 62/66) primarily registered for use in sheep and cattle. The most commonly used anthelmintics were a commercial combination of four anthelmintic drugs (abamectin, albendazole, levamisole and closantel), followed by BZs and MLs. Although targeted deworming was used on most respondents’ farms, very few were aware of strategic deworming and the issue of AR. The findings of this study provide valuable insights into the current gastrointestinal parasite control practices used by Australian dairy goat farmers. Although similar findings have been reported from Europe [15, 28, 30, 31, 34, 54, 55], South America [33] and Oceania [26, 27, 29], little is known about the parasite control practices used by goat farmers in Asian and African countries, where 90% of the global goat population is located. However, none of these studies comprehensively assessed the gastrointestinal parasite control practices in dairy goats, including the significance of these parasites in the dairy goat industry, and farmer’s knowledge and understanding of the diagnosis and control of gastrointestinal parasites.

We found that 74% (49/66) of respondents observed gastrointestinal parasite-related illnesses in their goats, mainly in the spring (32%; 34/105). These goat farmers had a good understanding of economically significant GINs and ranked *H*. *contortus*, *T*. *circumcincta* and *Trichostrongylus* spp. as ‘important’ for their animals. These GINs are the economically important internal parasites of sheep and goats globally and are also significant in Australian goats in regions of higher rainfall (i.e. > 380 mm) [36]. Although only 13% (21/155) of respondents sent faecal samples of dairy goats for parasite genus identification using the larval culture technique, their knowledge about the presence of different gastrointestinal parasites was comparable to those reported by McKenzie et al. [56] and Beveridge et al. [57]. An overwhelming majority of respondents (97%; 183/188) perceived gastrointestinal parasites could cause ill health and decreased milk production in dairy goats. Previous studies of Australian goats also found that GINs were a major problem for goat farmers [36–38]. In 2015, the annual cost of internal parasites (due to GINs) to the Australian goat industry was estimated at AUD 2.54 million [18]. Yet, our current understanding of the epidemiology of gastrointestinal parasites and their impact on Australian dairy goats in different agroclimatic regions is limited.

The range of antiparasitic products registered for treating goats in Australia is limited (*n* = 5 products) compared to those for sheep (https://wormboss.com.au/drenches-for-goats-using-products-correctly-and-legally/), with only one for dairy goats. This trend is expected to be similar in other regions of the world, where only a few or no antiparasitic drugs are registered for use in dairy goats due to the requirement of milk withdrawal periods, although some countries have a default milk withdrawal period (e.g. New Zealand, where this period is 35 days). In the present study, we found that 94% (62/66) of the respondents used antiparasitic drugs (see Table 5) and that the three most commonly used anthelmintics were a commercial combination of four anthelmintics (abamectin, albendazole, levamisole and closantel), BZs and MLs. This finding differs from those of three previous studies that surveyed Australian goat farmers [36–38], with the percentage of anthelmintic usage reported as 88% (172/195), 52% (16/31) and 85% (75/88), respectively. The discrepancies in anthelmintic usage between our study and those in these previous studies can be attributed to various factors, including the number of survey respondents and the target populations. Our study focused on dairy goat farmers with 66 respondents, while the previous studies surveyed larger or smaller populations of goat farmers involved in meat, dairy or fibre production. Moreover, differences in study periods, geographical locations, gastrointestinal parasite management practices and production systems among the surveyed populations may have influenced the choice and frequency of anthelmintic usage. Similar trends of substantial anthelmintic usage in goats have been documented globally, including 100% (number of survey respondents = 110) in Italy [15], > 85% (*n* = 847) in New Zealand [29], 95% (*n* = 73) in France [30], 89% (*n* = 57) in Greece [31], 84% (*n* = 284) in Brazil [33], 89% (*n* = 27) in Denmark [54], > 85% (*n* = 238) in Norway [55] and 97% (*n* = 37) in Portugal [58], with BZs and/or MLs being the first-choice anthelmintics. These studies demonstrate that the control of gastrointestinal parasites in goats has relied almost entirely on anthelmintics, presumably the main reason for the high prevalence of AR in goats [22].

In the present study, the majority of respondents used at least one anthelmintic drug that was not registered for goat use in Australia (see Table 5), consistent with the findings of Brunt et al. [38]. This finding is concerning because most respondents also kept sheep and cattle on their goat farms and these species shared the same paddocks as goats. Given that goats and sheep share similar GINs [59], there is a high risk of drug-resistant nematode species being transferred between sheep and goats [38], and Australia has the most drug-resistant GINs [60]. The presence of high levels of AR and the limited availability of registered products for goats in Australia pose a challenge for Australian goat farmers, leading them to select anthelmintic products registered for cattle and sheep. In addition, many anthelmintics have a ‘DO NOT USE’ on animals’ whose milk is used for human consumption statement on their label, which means that a veterinarian cannot prescribe them for these goats, even if they have data from overseas research on milk withholding periods. Consequently, there is a pressing need to register additional anthelmintics, such as combination products, and provide information on withdrawal periods for various milk and meat products.

The off-label use of anthelmintics in goats can pose further challenges when the dose rates vary. It is generally recommended to administer levamisole to goats at 1.5-fold the dose recommended for sheep [5, 61] and other anthelmintics at 1.5- to two fold the sheep dose [5]. We found that over half of the respondents (53%; 35/66) dewormed goats using 1.5-fold the dose recommended for sheep. Most of them (52%; 34/66) did not use a drenching gun for administering anthelmintics; when a drenching gun was used, only 20% (13/66) calibrated the gun before use. The dose rate for goats remains controversial as previous studies in Italy [15], New Zealand [27], France [30] and Ireland [34] reported using 1.5- to 2-fold the sheep dose. The administration of the correct dose of anthelmintics in goats also depends on accurately measuring the animal’s body weight. We found that only 20% (13/66) of Australian dairy goat farmers used the actual body weight and 29% (19/66) estimated it visually. A recent study reported that 89% (*n* = 110) of Italian dairy goat farmers calculated the dose of anthelmintics based on the estimated body weight of the heaviest goat in the herd [15], which is substantially greater than that in the present study (14%; 9/66). Similar findings have been reported from Denmark [28], France [30], Ireland [34] and Norway [55]. Using anthelmintics in goats at the dose rate recommended for sheep (e.g. 7/66 in this study) has significant implications for the development of AR, as goats metabolise anthelmintics faster than sheep, resulting in lower plasma concentrations and increased exposure of parasites to sub-therapeutic drug levels [59]. This can lead to more rapid development of AR in goats than sheep [62]. Accurate body weight measurement is recommended to prevent underdosing, which could help reduce the development of AR in goats.

To the authors’ knowledge, there is currently no registered topical or injectable anthelmintic product for use in goats in Australia (https://wormboss.com.au/drenches-for-goats-using-products-correctly-and-legally/). However, this study revealed that some farmers were using injectable (ivermectin) and/or pour-on preparations (see Table 5); this finding is in concordance with those of Brunt et al. [38]. Using topical or injectable antiparasitic products in goats is not only ineffective but also toxic to goats as goats have much less subcutaneous fat than sheep and cattle (https://wormboss.com.au). Additionally, unauthorised treatments in goats may have variable withdrawal periods, particularly due to their differing metabolism, leading to animals or animal products entering supply systems and posing a risk to public health [63]. In Australia, the legal use of antiparasitic drugs that are not registered for goat use requires an off-label veterinary prescription (https://wormboss.com.au). Our study showed that WormBoss and veterinarians were the two most trusted sources (108/236) for advice on the use of antiparasitic drugs in dairy goats (Fig. 7b). In the given circumstances, it is prudent that prescription-based use of antiparasitic drugs will help avoid underdosing which can lead to AR and pose risks to the safety of goats as well as the potential residues in milk or meat.

Using prescription-only anthelmintics with a targeted deworming strategy can reduce the selection pressure associated with AR by increasing the refugia population. In the present study, 67% (44/66) of respondents assessed worm burden using FECs. Most of these respondents (≥ 50%) were using targeted deworming for weaners and adult goats (Fig. 8), which is in concordance with previous studies on goats from Australia [36–38] but different from those from Italy [12], New Zealand [26], Ireland [34] and Brazil [33] where an interval-based deworming strategy was common. A previous Australian study reported that 72% (63/88) of goat farmers were using FECs to monitor worm burden [38]; this is similar to the 67% (44/66) of the respondents in this study who used FECs. In hot and humid zones in Australia (i.e. northern NSW and southern QLD) where *H*. *contortus* is highly prevalent, the FAMACHA© score could help apply targeted deworming in goats. However, only 13% (32/241) of respondents used this method to diagnose haemonchosis. Most respondents (77%; 51/66) in our study were unaware of AR on their farms, as only 23% (15/66) of them assessed the efficacy of anthelmintics, possibly due to a lack of information on the current status of AR in goats in Australia. AR in goats has been widely reported overseas [22], with very limited published reports available in Australia [64–67]. However, it is believed that goats and sheep typically harbour similar nematode species and are commonly found on the same premises (22 farms in this survey owned sheep and goats; Table 3). We assume that the AR situation in goats in Australia is comparable to that observed in sheep [68]. The increasing levels of AR for *H. contortus* is a major concern [69].

The MCA results revealed that better GIN control practices were adopted on dairy goat farms with semi-extensive or semi-intensive production systems, in contrast to farms with extensive production systems; the former constituted the majority of respondents in the present study. Additionally, farmers with good GIN control practices conducted FECs and employed targeted and/or strategic deworming strategies for adult dairy goats. Interestingly, farmers in this group also sought veterinary advice. However, they did not rotate antiparasitic drugs. This absence of drug rotation could be because farmers are likely actively seeking veterinary advice, and it is possible that veterinarians will not advise using antiparasitic drugs off-label, particularly in adult milking goats, as currently there is only one antiparasitic drug registered for use in Australian dairy goats. Although these findings are encouraging, dairy goat farmers using the extensive production system should adopt these best practices to make tailored on-farm decisions based on targeted deworming, which could slow the development of AR in GINs of goats.

Non-chemical methods, such as biological control and vaccination, can be used to control drug-resistant nematodes in goats. In the present study, 58% (43/76) of respondents used BioWorma®, 22% used COWP boluses and 4% used Barbervax® to control GINs and *H. contortus*, respectively. BioWorma® is a recently approved predaceous fungus in Australia, New Zealand and the USA, and is awaiting final approval in the European Union (www.bioworma.com). It contains chlamydospores of the nematophagus fungus *Duddingtonia flagrans* strain IAH1297, which is added to feed as a supplement to reduce larval contamination on pastures. Field trials in Australia have shown the effectiveness of BioWorma® in reducing the number of GIN larvae on pastures in goats (with an 86% reduction) and other livestock [70, 71]. The efficacy of *D*. *flagrans* against common GINs in goats has been assessed in various studies conducted worldwide [72–75]. Although COWP is approved for treating copper deficiency in goats in some regions of Australia, 22% (17/76) of respondents admitted to using COWP to control *H. contortus*. However, it is important to note that using COWP boluses for controlling *H. contortus* is considered an off-label use and should only be done under veterinary prescription. Additionally, it is recommended that COWP boluses are not used more than 4 times per year. We found that 4% (3/76) of respondents were using the Barbervax® vaccine. However, as the use of Barbervax® (https://barbervax.com/) in Australian goats is off-label, it requires further work as some promising findings have been published [76, 77]. Given that only one antiparasitic drug is now registered for use in dairy goats and current studies indicate that major nematode genera in goats (including *Haemonchus*, *Trichostrongylus*, *Teladorsagia*, *Oesophagostomum* and *Cooperia*) have already developed resistance to BZs, MLs and levamisole globally [22], it is advisable to utilise other methods of parasite control, such as BioWorma® and grazing management in goats. Furthermore, BioWorma® offers the added benefit of not requiring a milk withdrawal period, which is ideal for small dairy goat farmers in particular as it needs to be fed to the animals daily. In large dairy goat herds, it can be used strategically while introducing new goats in quarantine and at the periparturient rise period (www.goatvetoz.au).

In this study, 78% (51/66) of respondents perceived coccidia to be the second-most important gastrointestinal parasite of goats (Fig. 5) but only 52% (34/66) of them thought it to be a health problem in their goats, with almost half (16/34) of these using coccidiostats in kids and weaners. In a previous Australian study, 31% (61/195) of the respondents perceived coccidiosis as a problem occasionally, and 28% treated goats for coccidiosis [36]. The discrepancy between the current and previous studies could be due to differences in the study population and the number of survey respondents. In the earlier study [36], the majority of the respondents were meat (rangeland goat) farmers, and the respondent number was also higher (*n* = 195). To the contrary, the present study was solely based on 66 dairy goat farmers who kept kids and weaners in close proximity, thereby increasing the risk of coccidiosis due to increased stocking density.

Although coccidiosis is mainly a disease of kids, we found that only 16% (31/196) of farms reported using FECs in kids (Table 4). Furthermore, coccidia oocysts were identified on only 15% (27/180) of farms during FECs. In this study, spring was the main kidding season on most farms (64%; 42/66), and a significant proportion of respondents (76%; 50/66) were cleaning pens before kidding (Additional file 2: Figure S1), with most of them changing bedding materials 3 to 4 times or more than 4 times per month for the kids (Fig. 3). These husbandry practices could help reduce the occurrence of coccidiosis when correctly carried out. A previous survey study also indicated that respondents were aware of the hygiene requirements for the control of coccidiosis [36]. Ensuring minimal faecal contamination in the food and water is vital for intensively managed young goats. To achieve this, it is essential to elevate the feed and water troughs off the ground and prevent water leakage, as *Eimeria* oocysts can survive in moist conditions. A major challenge in intensive dairy goat production systems arises when kidding occurs multiple times over a year to maintain a consistent year-round milk supply. If the farmer uses the same pens constantly for successive batches or introduces newly born kids to a pen already housing older animals, the kids born later are immediately exposed to a heavy challenge and can show severe coccidiosis in the first few weeks of life [78]. Australian dairy goat farmers should be aware of pathogenic agents and host and environmental risk factors that may lead to a higher incidence of coccidiosis. For example, kids of a young age (i.e. < 6 months), high stocking densities, kidding outdoors, ingestion of a large dose of *Eimeria* oocysts, the presence of pathogenic species of *Eimeria* (*E. arloingi*,* E*. *ninakohlyakimovae*,* E*. *christenseni* and* E*. *caprina*), dirty conditions, intensive production system, stressful conditions and repeated use of rearing pens for different age groups of young goats are found to be associated with high levels of coccidiosis [36, 58, 78, 79].

Despite the novelty of this study, it is important to carefully interpret the results due to certain limitations. As of November 2023, the total number of dairy goat breeders registered with DGSA Ltd was 456 (personal communication). This includes breeders who are either out of practice or have ceased their membership, likely decreasing the size of our source population and increasing the proportion of the study population (respondents). Nevertheless, the response rate for this study was 14% (66/456), despite DGSA Ltd sending follow-up reminders to its members over a period of two months. This low response rate could introduce bias by either over-or-under representing certain results, potentially influenced by survey fatigue. This response rate (14%) is similar to a previous survey study with a response rate of 13% (195/1500) [36]. The most recent study acknowledged that estimating the response rate was difficult due to the use of electronic distribution [38]. This method is convenient and cost-effective, and provides fast access to many goat farmers [80]. However, considering the size of the Australian dairy goat industry, it should be noted that the respondents in our study represented a diverse range of farm sizes, production systems and levels of experience, and that their relative frequency was consistent with the frequency distribution of dairy goat farmers registered with DGSA Ltd (*n* = 456) with a PSI of 93% (Fig. 2). Additionally, selection bias may impact the interpretation of results. For example, there was no respondent from intensive production systems. Another potential source of bias could be missing data. In the present study, imputation, a commonly used method in health [81] and agricultural surveys [81], has been favoured over deletion or mean replacement for handling missing data [82].

## Conclusions

This study showed that nearly all (97%) of the surveyed dairy goat farmers perceived gastrointestinal parasites to be a cause of production losses in goats and *H*. *contortus* to be the most significant parasite. Australian dairy goat farmers commonly use anthelmintics registered for use in sheep and cattle and they seek veterinary advice for the use of these antiparasitic drugs. This study also highlights that the off-label use of anthelmintics at suboptimal dose rates might contribute to the development of AR in goats and poses risk to the industry regarding the potential to violate residues in goat products. The findings of this study could pave the way for tackling drug resistance in gastrointestinal parasites in goats and promote sustainable control of worms and coccidia through integrated parasite management.

## Supplementary Information

Additional file 1: Text S1. Questionnaire used in this study.

Additional file 2: **Table S1.** Variables selected for multiple correspondent analysis to determine the best gastrointestinal nematode control practices in Australian dairy goats. **Table S2.** Feed and water access at Australian dairy goat farms that responded to the survey.

Additional file 3: **Figure S1.** Percentage of respondents reporting the frequency of cleaning of pens before each kidding at Australian dairy goat farms. **Figure S2.** Percentage of respondents reporting their perceptions of gastrointestinal parasites diagnosed at Australian dairy goat farms. **Figure S3.** Percentage of respondents reporting the use of antiparasitic drugs in Australian dairy goats. **Figure S4.** Percentage of respondents reporting their perceptions about the main clinical signs of coccidiosis observed in kids.

## Data Availability

No datasets were generated or analysed during the current study.

## Abbreviations

AR: Anthelmintic resistance
DGSA: Dairy Goat Society of Australia Ltd
GINs: Gastrointestinal nematodes
MCA: Multiple correspondence analyses
