# Supplementary material for: Knowledge, attitudes and practices of Australian dairy goat farmers towards the control of gastrointestinal parasites
Source: Parasit Vectors. 2025 Jan 24;18:25. doi: 10.1186/s13071-024-06650-6 (PMC11761722; doi:10.1186/s13071-024-06650-6)
Supplement: Supplementary file 1 — Additional file 1: Text S1. Questionnaire used in this study. [file 13071_2024_6650_MOESM1_ESM.docx]

# Questionnaire to assess the gastrointestinal parasite control practices used by Australian dairy goat farmers

**Please complete all the questions by choosing one or more answers where appropriate. For**

**some questions, you will need to write your answer (s) in the space provided.**

1. Post Code
2. Farm area (hectares or acres) (Please indicate the correct unit) :
3. Grazing area (hectares) (Please indicate the correct unit) :


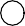

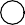

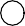

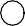


What is your role on the farm? Farm owner

Farm manager

Staff worker

Other

If you have answered 'Other' in previous question. Please specify 'Other':

1. What is your gender? Male

Female Other


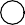

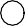

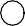

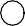


Prefer not to disclose.

1. For how long have you been working in the goat ≤1 year

industry? 1-5 years


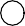

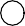

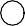

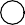

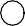


6-10 years

11-15 years

>15 years

1. Have you undertaken any degree/certificate and/or Yes

other formal training course(s) in agriculture/animal No science/goat farming?


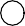

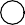


If yes, please specify:

1. How long has the property been used for goat farming? < 1 year

1-10 years


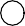

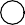

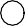

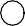

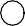

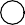


11-20 years

21-30 years

31-40 years

>40 years

1. What breed(s) of goats do you have on your farm? (Please choose all that apply)

Saanen Toggenburg British Alpine Anglo Nubian Australian Melaan Australian Brown Nigerian Dwarf Lamancha

Alpine Sable Other:

If you have answered 'Other' in previous question. Please specify 'Other'

1. Over the last 12 months, on average, how many goats have you had on your farm in the following categories? (You can estimate if you do not have any record of exact numbers in each category.) Please add '0' if you do not have an animal in the given category.

Kids (≤ 6 months)

Weaners (≥ 6 months - 1 year)

Milkers/does

Bucks

10**.** What is the approximate average covered area space Less than 0.5 m2/goat allowance per housed goat at your farm? 0.5 - 1.0 m2/goat

1.0 - 1.5 m2/goat


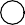

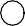

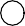

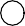

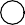

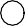

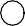


1.5 - 2.0 m2/goat

2.0 - 2.5 m2/goat

2.5 - 3.0 m2/goat

More than 3.0 m2/goat

1. For indoor housing of does, what bedding material do you use? (Please choose all that apply.)

Slatted floor: plastic, wood or expanded metal Solid floor with straw or wood shavings

Other

If you have answered 'Other' in previous question. Please specify 'Other':

1. How would you describe your goat farm?
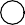
 Extensive (animals graze on a large area, with a low animal density)

Intensive indoor (animals do not graze and are kept housed and fed forage/supplementary feed)


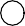


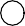
 Semi-intensive (an intermediate compromise between extensive and intensive systems, but usually with controlled grazing)


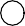
 Semi-extensive (animals are grazed and supplemented with crop residues and supplementary feed)


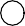
 Other

If you have answered 'Other' in previous question. Please specify 'Other':

1. In which season, over the last five years, have most Summer

of the kids been born? Autumn


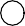

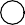

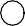

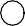

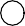


Winter Spring

Non-­seasonal


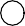

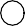

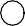

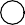

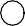


14**.** How often do you clean pens before each kidding? Always Usually Sometimes Rarely Never

1. What is the main source of the colostrum used at your farm? (Please choose all that apply.)

Fresh from does in your own herd

Frozen but collected from does in your own herd Cow colostrum as a substitute

Colostrum supplements Colostrum replacers Milk replacer

Other

If you have answered 'Other' in previous question. Please specify 'Other':

1. What method(s) do you use to feed milk to kids? (Please choose all that apply.)

Naturally (i.e. from the doe) Bottle feeding

Open vessel feeding Self-feed teat feeding

Electronic automatic milk feeder Other

If you have answered 'Other' in previous question. Please specify 'Other':

1. What is the average weaning age of kids on your farm? < 8 weeks

8 - 10 weeks


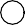

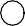

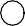

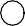


> 10 weeks Other

If you have answered 'Other' in previous question. Please specify 'Other':

1. How often do you change bedding?

Kids Weaners Does Bucks

Less than 1 time

/month


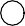


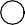

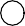

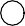


1 - 2 times /month 3- 4 times /month More than 4 times

/month


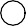

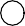

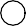


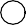

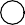

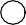

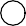

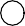

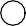

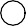

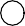

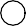


1. What type of feed do you provide to weaners and does on your farm?

Weaners Does

Silage (Grass, Maize)

Hay (Grass, Straw, Barley, WGrheeeant)Crops

By-products (Dried Brewer's grain, Sugar Beet Pulp, Maize Gluten)

Pellets Other

If you have answered 'Other' for weaners in previous question. Please specify 'Other':

If you have answered 'Other' for does in previous question. Please specify 'Other':

1. Do you usually provide any supplementary feed to your Yes goats? No

Not applicable


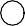

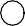

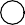


If yes, why do you provide supplementary feed to your goats? (Please choose all that apply.)

Insufficient feed on offer Poor quality of pasture

Physiological status of goats (e.g., lactating does)

Finisher feed for kids before sending to an abattoir

Poor condition of animals Other

If you have answered 'Other' in previous question. Please specify 'Other':

1. Do your goats have access to any surface water from any of the following? (Please choose all that apply.)

No access to surface water other than that provided artificially.

Ponds

Irrigation ditches Streams/creeks Other

If you have answered 'Other' in previous question. Please specify 'Other':

1. What are the other livestock species kept on your farm? (Please choose all that apply.)

None Cattle Sheep Pigs Alpacas Other

If you have answered 'Other' in previous question. Please specify 'Other':

1. Do goats on your farm share paddocks with other Yes


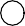

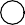

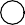


livestock species? No

Occasionally

If yes, do you practice any of the following at your farm? (Please choose all that apply.)

Rotational grazing with other livestock (moving livestock through a series of paddocks so when they have finished grazing the last paddock in the series, the first paddock has recovered to allow the rotation to recommence).

Co-grazing (i.e., more than one livestock species graze in the same paddock at the same time).

If goats on your farm share paddocks, which other species do they share the paddocks with?

(Please choose all that apply.)

Rotational grazing Co-grazing

Cattle
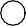

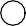


Sheep
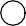

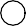


Alpacas
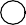

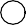


Horses
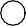

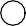
 Pigs
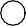

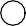
 Other
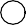

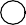


If the answer to the previous question was "Other" please specify (Rotational grazing):

If the answer to the previous question was "Other" please specify (Co- grazing):

If you practice rotational or co grazing of goats with Unweaned calves

cattle, what is the age category of the cattle? Weaned calves < 12 months Cattle between 1-2 years Cattle >2 years


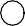

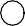

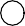

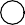


1. In the last 12 months, have any of your goats been Yes

suspected of illness due to parasites/ worms? No


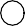

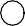

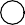


Don't know

1. Which of the following signs have you observed in goats with illness suspected to be related to parasites? (Please choose all that apply.)

Scouring/diarrhoea

Perineal soiling (presence of sticky faeces near the anus causing dermatitis and discharge of a brown fluid)

Weight loss

Anaemia (pale mucous membrane of the eyes and mouth)

Weakness Bottle jaw Rough coat

High FAMACHA score* (FAffa MAlan CHArt Score) Death

Other

(*FAMACHA score: This system uses an eye-color chart developed as a guide to identify anemia, a symptom of parasite infection. This involves checking the mucus membranes inside of the lower eyelid (conjunctiva) and comparing it to colours on a FAMACHA card. The colour of the conjunctiva reflects the amount of red blood cells in the animal.)

If you have answered 'Other' in previous question. Please specify 'Other':

1. In which seasons(s) do you most often observe illness/production losses due to internal parasites in goats on your farm? (Please choose all that apply.)

Spring Summer Autumn Winter

Non seasonal Never

1. Based on your experience, which of the following groups of goats are susceptible to the risk of

worms/ internal parasites at your farm? (Please choose any that apply.

Kids (≤ 6 months) Weaners (~1 year) Does/milkers Bucks

Never Rarely Sometimes Usually Always


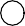

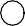

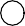

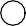

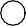


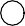

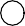

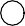

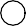

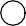

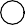

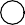


1. Which parasites have been identified previously on your farm, if any? (Please choose all that apply.)

Barber's pole worm (Haemonchus contortus) Black scour worm (Trichostrongylus spp.)

Brown stomach worm (Teladorsagia circumcincta) Nodule worm (Oesophagostomum columbianum) Small bowel worm (Oesophagostomum venulosum) Large-mouthed bowel worm (Chabertia ovina) Small Lungworm (Muellerius capillaris)

Enteric coccidia (Eimeria spp.) Liver fluke (Fasciola hepatica) Tapeworm (Moniezia sp.)

Other

Do not know

If you have answered 'Other' in previous question. Please specify 'Other':

1. Which parasites, if any, do you consider important in your flock or for goat health? (Please

choose all that apply.)

Barber's pole worm (Haemonchus spp.)

Black scour worm (Trichostrongylus spp.)

Brown stomach worm (Teladorsagia circumcincta)

Small lungworm (Muellerius capillaris)

Enteric coccidia (Eimeria spp.) Liver fluke (Fasciola hepatica) Tapeworm (Moniezia sp.)

Important Not important Unsure

1. What are the most important negative impacts that internal parasites have on your goat farm? (Please choose all that apply.)

Increased morbidity and mortality

Reduced appetite, resulting in debilitated animals that are more susceptible to other diseases Transmission of diseases from one goat to another as some parasites can also act as vectors (carriers)

Reduced grazing behavior or agitation Reduced growth rates

Reduced reproductive rates

Reduced income through condemnation of carcass parts at slaughter

Reduced milk production None

1. What are the major methods of possible internal parasite transmission on your farm? (Please choose all that apply.)

Suboptimal hygienic conditions Contamination of feed by fecal matter Overgrazing

Higher stocking density

Incorrect use of anti-parasitic drugs Malnutrition

Other

If you have answered 'Other' in previous question. Please specify 'Other':

1. When an internal parasite-related illness had been Yes suspected in your goats, was veterinary advice sought No when making the diagnosis and treatment decisions?
2. Which of the following method(s) are/were used at your farm to diagnose worms/ internal parasites in goats? (Please choose all that apply.)

None Clinical signs

Observation of worms in the faeces Faecal (worm) egg count Faecal/larval culture

Postmortem examination (autopsy) Blood/serum examination

Other

If you have answered 'Other' in previous question. Please specify 'Other':

1. Do you perform faecal (worm) egg counts for goats on Yes your farm? No

Don't know

1. What is your purpose of performing faecal egg counts for your goats? (Please choose all that apply.)

Monitoring of worm burden

Diagnosis of illness due to worms/ parasites Assessing the effectiveness of dewormers For deciding which goats need deworming Other

If you have answered 'Other' in previous question. Please specify 'Other':

1. If you do monitor faecal egg counts of goats on the farm, which goats are sampled? (Please choose all that apply.)

Kids (≤ 6 months) Weaners (~ 1 year) Does/milkers Wethers

Bucks

Aged (> 6 years)

1. If you use faecal egg counts at your farm, where are General diagnostic laboratory

the samples analysed? Commercial worm egg counting services (e.g. Worm count etc.)

Veterinary clinic

On-farm by the farm staff

1. Do you use antiparasitic drugs Yes

(dewormers/drenches/anthelmintics/coccidiostats) for No the control of parasites on your farm?

1. Which preparation(s) of antiparasitic drugs do you use? (Please choose all that apply.)

Oral Injectable Pour­-on

Oral and injectable Oral and pour-­on

Oral, injectable and pour-­on Other

If you have answered 'Other' in previous question. Please specify 'Other':

1. When do you give first dose of antiparasitic drugs to Pre weaning goats born at your farm? At weaning

Post weaning Other

If you have answered 'Other' in previous question. Please specify 'Other':

1. How do you decide about a parasite control schedule for goats on your farm? (Please choose all that apply.)

Follow veterinarian's recommendation Based on faecal egg count results

According to a professionally written worm control plan

Follow a friend's/colleague's recommendation My own knowledge on goat worms

Goat production manual

Ask for advise on social media Other

If you have answered 'Other' in previous question. Please specify 'Other':

1. What is your main source of advice on parasite control in goats? (Please choose all that apply.)

Veterinarians

Sales representatives Other goat farmers Scientific journals Manufacturer's instructions

Newsletters written by veterinarians or breed society newsletters

Wormboss Paraboss

State department websites Facebook goat groups Other

If you have answered 'Other' in previous question. Please specify 'Other':

1. Which of the following strategies best describes how you decide to deworm goats on your farm?

Interval

Dosing (e.g. drenched at set intervals)

Strategic

Dosing (e.g.drenched at particular times of year to target different types of parasites)

Targeted

Dosing (e.g. drenched based on the results of faecal egg counts)

Goats in this

age group are never drenched on my farm.

Goats are

drenched at a time that is most convenient.

Other

1. Kids (up to 6 month)
2. Yearlings (> 6 months-1 year)
3. Adults (>1 year)

If you have answered 'Other' in previous question. Please specify 'Other' (Kids):

If you have answered 'Other' in previous question. Please specify 'Other' (Yearlings):

If you have answered 'Other' in previous question. Please specify 'Other' (Adults):

1. In which month(s) do you typically administer dewormers to your goats? (Please choose all that apply)

January February March April May June

July August September October November December

Not following any fixed schedule

1. Please provide a list of dewormers you have used in the last five years.
2. What other methods do you use to control GI parasites Copper Oxide Wire Particles boluses of goats on your farm? (Please choose all that apply) Barbervax

Bioworma Other

If you have answered 'Other' in previous question. Please specify.

1. What dose rate do you use to administer antiparasitic Dose recommended for sheep drugs to your goats? Dose recommended for cattle

One­ and ­a­ half times of the dose recommended for sheep

Two times of the dose recommended for sheep Three times of the dose recommended for sheep Other

If you have answered 'Other' in previous question. Please specify 'Other':

1. If you use a drenching gun, how often do you calibrate I do not use a drenching gun

it? My drenching gun does not require calibration

Occasionally

Every time before I use it Never

1. What criterion do you use to calculate the dose of an Actual body weight of an animal

antiparasitic drug for goats? A visual estimate of the body weight of an animal An average body weight of all animals on your farm An estimate of the body weight of the heaviest animal

Actual body weight of the heaviest animal in the herd

Heart girth table Other

If you have answered 'Other' in previous question. Please specify 'Other':

1. How often do you rotate/change the antiparasitic No rotation

drug(s) at your farm? Annually

Every two years Every three years Every four years Every five years

1. Have your goats been diagnosed with coccidiosis Yes

previously? No

1. Have you observed diarrhoea/perineal soiling or reduced weight gain in kids up to 8 weeks of age?

In 0-20% In 21-40% In 41-60% In 61-100% Not seen

1. Diarrhoea/perineal soiling
2. Reduced weight gain
3. Tenesmus (diarrhoea with

'straining' )

1. Which chemical products have you used to control coccidiosis?
2. Do you provide coccidiostat (antiprotozoal agent that Yes

acts upon Coccidia parasites) for goats at your farm? No

1. To which of the following groups of goats do you provide coccidiostats? (Please choose all that apply)

Kids (≤ 6 months) Weaners (~ 1 year) Does/milkers Pregnant does Bucks

Aged (> 6 years)

1. Have your goats been tested for resistance to Yes

antiparasitic drug(s) in the last five years? No

If yes, how was the resistance to dewormers tested? My veterinarian performed faecal egg counts before

and after the administration of antiparasitic drug(s)

I performed faecal egg counts before and after the administration of antiparasitic drug(s)

I do not know

1. Which dewormers are no longer effective on your property (i.e. parasites have become resistant)?
2. What types of compounds/remedies (i.e., other than Not using remedies other than commercially drugs or chemicals that are registered for any available drugs

species), if any, do you use to control parasites in Herbal remedies

your goats? (Please choose only one option.) Other

If you have answered 'Other' in previous question, please specify 'Other':

If you have answered 'Herbal remedies' in previous question, please specify:
