# Supplementary material for: Knowledge, attitudes and practices of Australian dairy goat farmers towards the control of gastrointestinal parasites
Source: Parasit Vectors. 2025 Jan 24;18:25. doi: 10.1186/s13071-024-06650-6 (PMC11761722; doi:10.1186/s13071-024-06650-6)
Supplement: Supplementary file 2 — Additional file 2: Table S1. Variables selected for multiple correspondent analysis to determine the best gastrointestinal nematode control practices in Australian dairy goats. Table S2. Feed and water access at Australian dairy goat farms that responded to the survey. [file 13071_2024_6650_MOESM2_ESM.docx]

**Table S1** List of variables selected for multiple correspondent analysis (*n =* 66) to determine the best gastrointestinal nematode control practices in Australian dairy goats

| Variable | Levels | Response % (counts) |
| --- | --- | --- |
| Role on the farm | Farm owner | 94 (62) |
|  | Farm manager | 5 (3) |
|  | Staff worker | 1 (1) |
| Gender | Female | 91 (60) |
|  | Male | 8 (5) |
|  | Prefer not to disclose | 1 (1) |
| Experience of goat farming (years) | 1-5 | 38 (25) |
|  | 6-10 | 18 (12) |
|  | 11-15 | 12 (8) |
|  | >15 | 32 (21) |
| Formal education | No | 80 (53) |
|  | Yes | 20 (13) |
| Herd size | Small | 36 (24) |
|  | Medium | 56 (37) |
|  | Large | 8 (5) |
| Production system | Semi-extensive | 65 (43) |
|  | Extensive | 23 (15) |
|  | Semi-intensive | 12 (8) |
| Supplementary feed | Yes | 96 (63) |
|  | No | 3 (2) |
|  | Not applicable | 1 (1) |
| Co-grazing management | Yes | 58 (38) |
|  | No | 42 (28) |
| Veterinary advice sought | Yes | 55 (36) |
|  | No | 45 (30) |
| Use of FEC | Yes | 67 (44) |
|  | No | 32 (21) |
|  | Do not know | 1 (1) |
| Use of antiparasitic drugs | Yes | 94 (62) |
|  | No | 6 (4) |
| Veterinary consultation on parasite control | Yes | 76 (50) |
|  | No | 24 (16) |
| WormBoss consultation on parasite control | Yes | 70 (46) |
|  | No | 30 (20) |
| Targeted or strategic deworming of adult goats | Yes | 70 (46) |
|  | No | 30 (20) |
| Targeted or strategic deworming of weaners | Yes | 68 (45) |
|  | No | 32 (21) |
| Dose calculation for antiparasitic based on the actual body weight of individual goats and/or the heaviest goat in the herd | No | 77 (51) |
|  | Yes | 23 (15) |
| Rotation between anthelminthics classes | Yes | 55 (36) |
|  | No | 45 (30) |
| Anthelmintic resistance tested (using FECRT) | No | 77 (51) |
|  | Yes | 23 (15) |

FEC, Faecal egg count; FECRT, FEC reduction test

**Table S2** Feed and water access at Australian dairy goat farms that responded to the survey

| Question (responses) | Levels | Percentage (counts) |
| --- | --- | --- |
| Main source of colostrum for kids (*n* = 81)^a^ | Fresh from does in own herd | 78 (63) |
|  | Frozen but collected from does in their own herd | 15 (12) |
|  | Milk replacers | 7 (6) |
|  |  |  |
| Method(s) used to feed milk for kids (*n* = 113)^a^ | Bottle /lambar feeding | 47 (53) |
|  | Naturally (from the doe) | 41 (46) |
|  | Self- feed teat feeding | 10 (11) |
|  | Open vessel feeding | 3 (3) |
|  |  |  |
| Type of feed for weaners (*n* =179)^a^ | Roughage (hay, chaff, bran, grass, straw) | 37 (66) |
|  | Concentrate (barley, wheat, pellets, cracked grains) | 35 (62) |
|  | Browse and grazing (green crops, shrubs, trees, free grazing etc.) | 17 (31) |
|  | Concentrate and roughage | 8 (15) |
|  | Silage | 2 (4) |
|  | Other | 1 (1) |
|  |  |  |
| Type of feed for does (*n* =175)^a^ | Roughage | 37 (64) |
|  | Concentrate | 37 (64) |
|  | Browse and grazing | 17 (29) |
|  | Concentrate and roughage | 7 (13) |
|  | Silage | 2 (4) |
|  | Other | 1 (1) |
|  |  |  |
| Supplementary feed (*n* = 66) | Yes | 95 (63) |
|  | No | 3 (2) |
|  | Not applicable | 2 (1) |
|  |  |  |
| Reason for supplementary feeding (*n* = 110) ^a^ | Physiological status of goats (e.g., lactating does) | 44 (48) |
|  | Poor quality of pasture | 18 (20) |
|  | Insufficient feed on offer | 17 (19) |
|  | Poor condition of animals/maintain good health | 9 (10) |
|  | Finisher feed for kids before sending them to an abattoir | 6 (6) |
|  | Weather (raining or too warm) | 2 (2) |
|  | Grazing unavailable | 1 (1) |
|  | Other ^b^ | 4 (4) |
|  |  |  |
| Access to surface water (*n* = 70)^a^ | No access to surface water other than that provided artificially | 71 (50) |
|  | Ponds and/or dam | 17 (12) |
|  | Streams or creeks | 12 (8) |

^a^ These questions had more than one answers

^b^ For show animals (*n* = 1), to keep the weaners/other goats friendly and easier to manage (*n* = 2) and mineral lick blocks (*n* = 1).
