# Supplementary material for: Knowledge, attitudes and practices of Australian dairy goat farmers towards the control of gastrointestinal parasites
Source: Parasit Vectors. 2025 Jan 24;18:25. doi: 10.1186/s13071-024-06650-6 (PMC11761722; doi:10.1186/s13071-024-06650-6)

**Legends of supplementary Figures**

**Fig. S1.** Percentage of respondents (*n* = 66) reporting the frequency of cleaning of pens before each kidding at Australian dairy goat farms

**Fig. S2.** Percentage of respondents (*n* = 66) reporting their perceptions of gastrointestinal parasites diagnosed at Australian dairy goat farms.

**Fig. S3.** Percentage of respondents (*n* = 66) reporting the use of antiparasitic drugs in Australian dairy goats.

**Fig. S4.** Percentage of respondents (*n* = 66) reporting their perceptions about the main clinical signs of coccidiosis observed in kids.


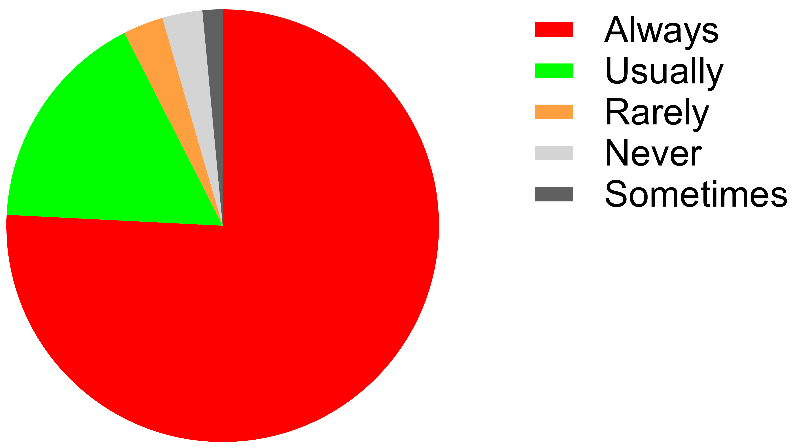


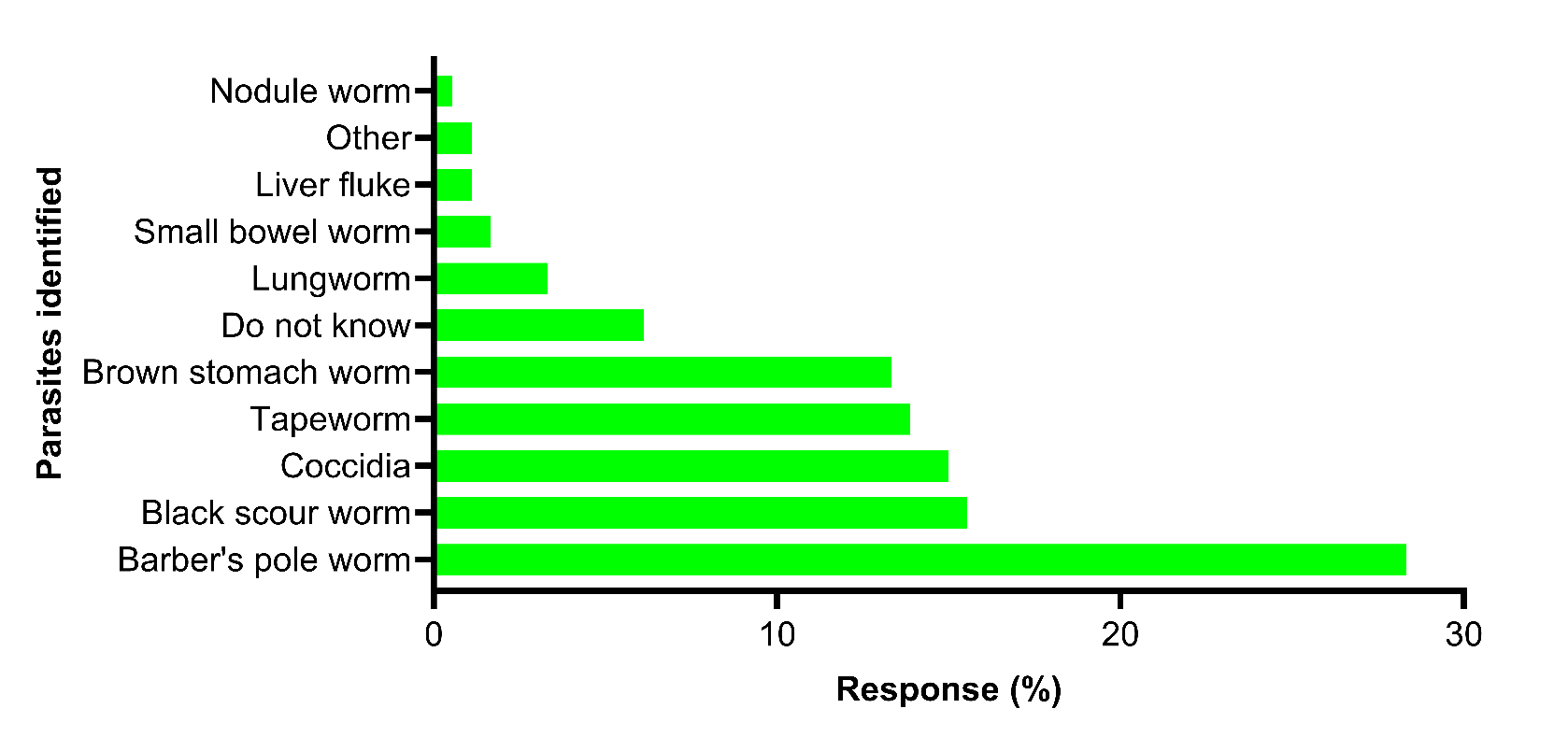


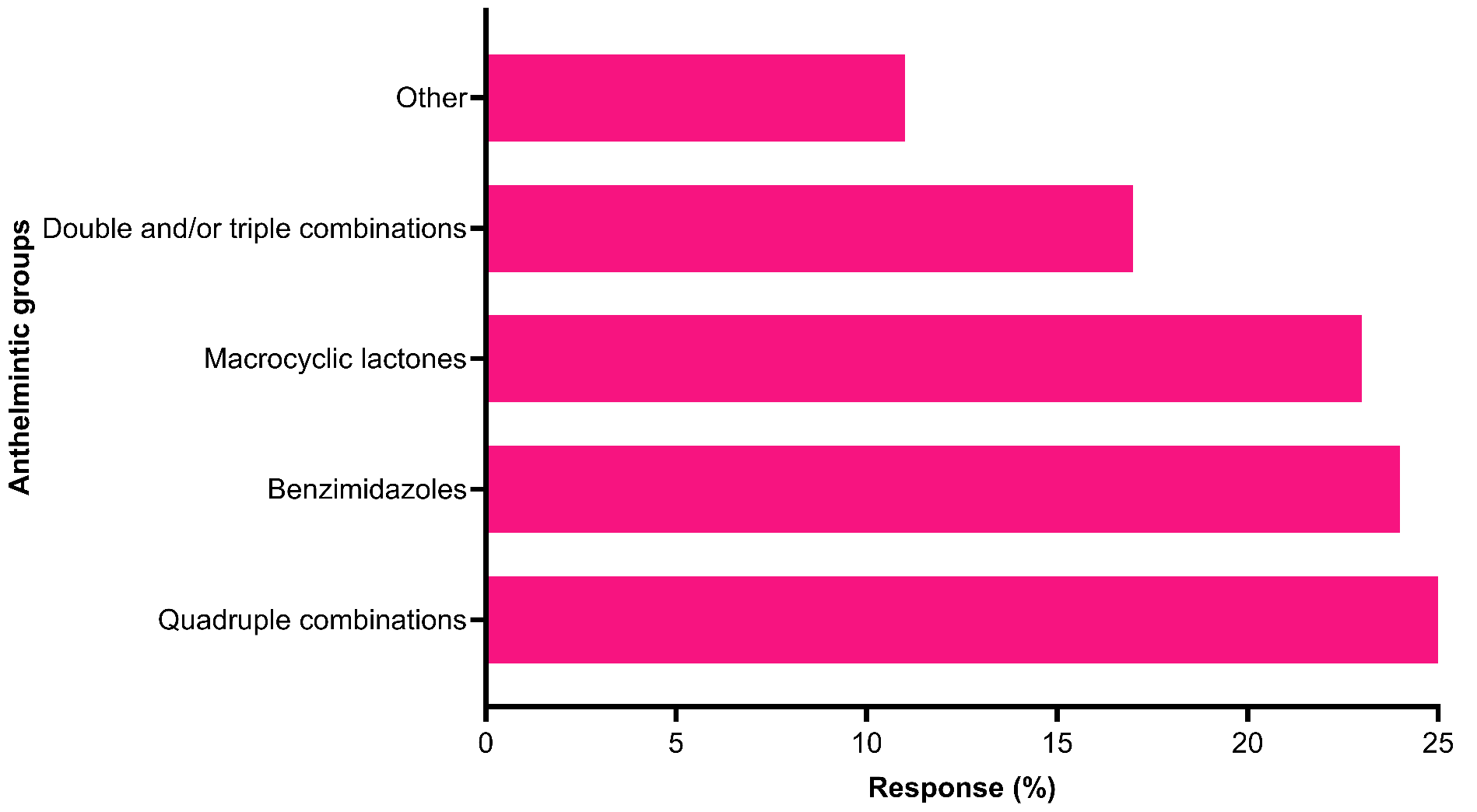


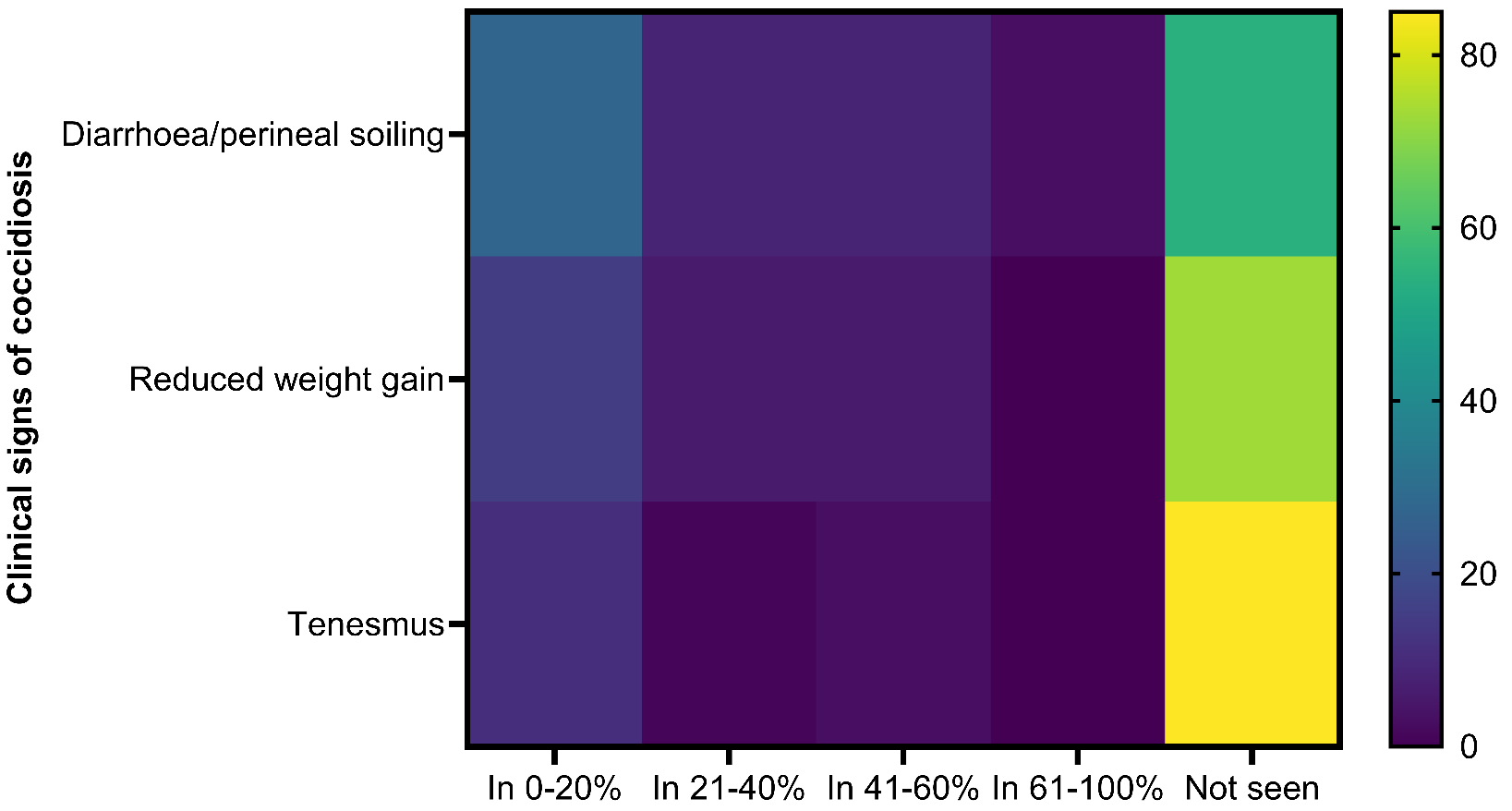

Supplement: Supplementary file 3 — Additional file 3: Figure S1. Percentage of respondents reporting the frequency of cleaning of pens before each kidding at Australian dairy goat farms. Figure S2. Percentage of respondents reporting their perceptions of gastrointestinal parasites diagnosed at Australian dairy goat farms. Figure S3. Percentage of respondents reporting the use of antiparasitic drugs in Australian dairy goats. Figure S4. Percentage of respondents reporting their perceptions about the main clinical signs of coccidiosis observed in kids. [file 13071_2024_6650_MOESM3_ESM.docx]
